# A novel integrated non-targeted metabolomic analysis reveals significant metabolite variations between different lettuce (*Lactuca sativa*. L) varieties

**DOI:** 10.1038/s41438-018-0050-1

**Published:** 2018-06-25

**Authors:** Xiao Yang, Shiwei Wei, Bin Liu, Doudou Guo, Bangxiao Zheng, Lei Feng, Yumin Liu, Francisco A. Tomás-Barberán, Lijun Luo, Danfeng Huang

**Affiliations:** 1 School of Agriculture and Biology, Shanghai Jiao Tong University, Key Laboratory of Urban Agriculture, Ministry of Agriculture, Shanghai, 200240 China; 2 Shanghai Agrobiological Gene Center, Shanghai, 201106 China; 3 Research Group on Quality, Safety and Bioactivity of Plant Foods, Center for Applied Soil Science and Biology of the Segura, the Spanish National Research Council, (CEBAS-CSIC), Murcia, 30100 Spain; 4 Key Laboratory of Urban Environment and Health, Institute of Urban Environment, Chinese Academy of Sciences, Xiamen, 361021 China; 5 University of Chinese Academy of Sciences, Beijing, 100049 China; 6 Instrumental Analysis Center, Shanghai Jiao Tong University, Shanghai, 200240 China

## Abstract

Lettuce is an important leafy vegetable that represents a significant dietary source of antioxidants and bioactive compounds. However, the levels of metabolites in different lettuce cultivars are poorly characterized. In this study, we used combined GC × GC-TOF/MS and UPLC-IMS-QTOF/MS to detect and relatively quantify metabolites in 30 lettuce cultivars representing large genetic diversity. Comparison with online databases, the published literature, standards as well using collision cross-section values enabled putative identification of 171 metabolites. Sixteen of these 171 metabolites (including phenolic acid derivatives, glycosylated flavonoids, and one iridoid) were present at significantly different levels in leaf and head type lettuces, which suggested the significant metabolomic variations between the leaf and head types of lettuce are related to secondary metabolism. A combination of the results and metabolic network analysis techniques suggested that leaf and head type lettuces contain not only different levels of metabolites but also have significant variations in the corresponding associated metabolic networks. The novel lettuce metabolite library and novel non-targeted metabolomics strategy devised in this study could be used to further characterize metabolic variations between lettuce cultivars or other plants. Moreover, the findings of this study provide important insight into metabolic adaptations due to natural and human selection, which could stimulate further research to potentially improve lettuce quality, yield, and nutritional value.

## Metabolic analysis: Not all lettuces are equal

Lettuces of different types show distinct variations in metabolic composition, which may have significance for their nutritional value. Lettuce (*Lactuca sativa*) has been cultivated for over 4000 years, resulting in a huge diversity of morphological types. Using a powerful combination of analysis techniques, an international team led by Francisco Tomas-Barberan at the Centre for Applied Soil Science and Biology of the Segura, Murcia, Spain, and Lijun Luo and Danfenge Huang of Shanghai Agribiological Gene Center, China, explored the chemical differences between thirty types of lettuce including leafy, butterhead, iceberg and romaine varieties. Their comprehensive ‘metabolomic’ approach identified significant differences in metabolism between leafy types and the three other, ‘head’ types, with the leafy varieties having higher concentrations of health-promoting compounds such as antioxidants. Their approach could in future be applied to other types of crops.

## Introduction

Lettuce (*Lactuca sativa* L.), an important vegetable crop that is consumed worldwide, is one of the *Asteraceae* species, which originated in Ancient Egypt, and was cultivated as early as 2500 BCE^1^. Both natural and human selection have generated a variety of lettuce cultivars with huge genetic diversity. Leaf-edible lettuce cultivars are classified as leafy and head types based on their morphological features; there are three head types: romaine, iceberg, and butterhead. Lettuce cultivars have significantly different genetic, phenotypic and commercial characteristics (e.g., stress resistance, nutritional quality). To date, many considerable efforts had been made to explore the insights of genomics^2^ and transcriptomics^3^ of *Lactuca sativa*. However, there is limited knowledge of the chemical composition of individual cultivars and its effect on plant development and adaption.

Daily consumption of lettuce has been shown to promote human health and reduce the incidence of a number of chronic diseases; these effects have been attributed to the presence of bioactive phytochemicals in lettuce^4^. As metabolism is strongly affected by genetic factors, different lettuce cultivars are likely to have huge metabolite diversity. Thus, in an effort to improve human health, assessment of the metabolic profiles of a variety of leaf and head type lettuces may help to identify the most nutritionally valuable varieties.

Metabolomics is a comprehensive metabolic profiling approach that enables analysis of a wide range of metabolite classes simultaneously in a non-biased manner^5^. Several techniques have been employed to qualitatively assess the phytochemicals present in lettuce, including gas chromatography–mass spectrometry (GC–MS), liquid chromatography–mass spectrometry (LC–MS) and nuclear magnetic resonance (NMR)^6–13^; these techniques rely on accurate comparison of the mass-to-charge ratio (*m/z*) and retention time (RT) for each metabolite with known standards. For example, Lee et al.^6^ quantified nine types of phytotoxic organic acids in lettuce using GC/MS. Abu-Reidah et al.^12^ and Viacava et al.^7^, putatively identified 171 and 115 compounds in lettuce cultivars using ultra-high-performance liquid chromatography-quadrupole-time-of-flight mass spectrometry (UPLC-QTOF-MS), respectively. These metabolites included amino acids, peptides, organic acids, alkaloids, terpenoids, phenolic compounds, and lipids. Using UPLC-ESI-QTOF-MS, García et al.^8,13^, explored the metabolites in iceberg and romaine lettuce, as well as the metabolic changes that occurred during enzymatic browning. However, due to the complexity of the plant metabolome, accurate identification and quantification of compounds remain a huge challenge^14,15^.

In this regard, the use of advanced analytical platforms, such as comprehensive two-dimensional gas chromatography/time-of-flight mass spectrometry (GC × GC/TOF-MS) or ultra-performance liquid chromatography-ion mobility spectrometry-quadrupole time-of-flight mass spectrometry (UPLC/IMS/QTOF-MS) have emerged as one of the most sensitive tools for molecular characterization^16,17^. Two-dimensional GC × GC (2D-GC × GC) coupled with TOF can improve co-effluent separation, chromatographic resolution and analyte detection compared to one-dimensional-gas chromatography (1D-GC)^16^. Ion mobility (IMS)-based UPLC can distinguish the isomeric structures of co-effluent metabolites based on their drifting time through the buffer gas and generates collision cross-section (CCS) data to aid metabolite identification, making this technique more robust and reproducible^17^. Using GC × GC-TOF/MS, Hurtado et al.^18^ identified 50 lettuce metabolites related to sugar metabolism, the citric acid cycle and the pentose phosphate pathway. We previously applied a methodology based on UPLC/IMS/QTOF-MS to characterize the secondary metabolites present in lettuce, and putatively annotated 35 types of polyphenols^19,20^. However, in spite of the great advances in knowledge provided by these studies, a comparison of primary and secondary metabolite diversity between the leaf and head (romaine, iceberg, and butterhead) types of lettuce has not yet been reported.

Therefore, the present study aimed to provide a global view of the metabolite diversity and metabolic pathway variations in a collection of 30 different lettuce cultivars, which represents large genetic diversity. Using GC × GC-TOF-MS and UPLC-IMS-QTOF-MS, we detected and quantified the metabolites in the 30 lettuce cultivars and further suggest the biological significance of the metabolic differences using a variety of metabolic network analysis techniques.

## Materials and methods

### Plant materials and morphological characteristics assessment

This study was based on a lettuce cultivar collection that includes 18 leaf and 12 head (including six romaine, three iceberg and three butterhead) lettuce cultivars. Lettuce seeds were randomly arranged and sown in seedling trays in the nursing substrate (1:1 turf: perlite, *v:v*) during the autumn of 2016, and germinated and cultivated in a greenhouse at Shanghai Academy of Agriculture Sciences (30.89°N, 121.39°E). After 2 weeks, lettuce seedlings were transplanted to 32 holes of seedling trays. During the cultivation, the temperature was maintained at 20 ± 3 °C during the day and 13 ± 2 °C at night and light irradiance was 180–220 μmol m^−2^ s^−1^ during the 12 h photoperiod. Twenty-eight-day-old lettuce seedlings (five leaves and one bud) were collected, flash frozen in liquid nitrogen and stored at −80 °C until analysis. The morphological characteristics assessment of lettuce (80 days after field sowing) was performed in spring of 2016 according to the previous study^21^.

### Metabolite profiling

#### GC × GC-TOF-MS analysis

Lettuce leaf tissue (0.2 g) was ground into a fine powder in liquid nitrogen and extracted in 1 mL ice-cold methanol: chloroform solution (3:1, v:v), as described previously^19^.

The two-dimensional gas chromatography/time-of-flight mass spectrometry system (Pegasus 4D; PerkinElmer Inc., Waltham, MA, USA) consisted of an Agilent 7890 GC and a TOF-MS. Analytes were initially separated on a non-polar DB-5MS column (30 m × 0.25 mm × 0.25 μm, Agilent J&W Scientific, Folsom, CA, USA) based on vapor pressure into numerous adjacent small fractions, then each fraction was subsequently re-injected onto a DB-17MS column (2 m × 0.10 mm × 0.10 μm; Agilent J&W Scientific) for secondary separation. A modulator was used to connect two GC columns, which could transfer eluates from DB-5MS column into DB-17MS column to facilitate complete separations. Sample volume was 1 μL; inlet temperature, 280 °C; carrier gas, helium; flow rate, 1 mL min^−1^; GC oven temperature was 90 °C for the first 1 min, then increased to 220 °C at 4 °C min^−1^, 300 °C at 25 °C/min, and held for 10.8 min. Secondary oven temperature was 5 °C higher than the primary oven. Modulation period was 4.0 s, and the temperature of modulator was 5 °C higher than the secondary oven. The MS was performed in electron impact ionization mode at 70 eV, with scanning from 33 to 600 m/z at 50 spectra s^−1^ at an acquisition voltage of 1500 V, electron impact ionization energy of 70 eV and acquisition voltage of 1700 V. Transfer line and ion source temperatures were 270 °C and 220 °C. Before any samples were processed, 1 μL of a fatty acid methyl ester mixture (C6-C24) was analyzed. Furthermore, quality control (QC) samples (a mixture of all samples to be analyzed) were run at the beginning, middle, and end of each batch.

#### UPLC-IMS-QTOF-MS/MS analysis

For UPLC-IMS-QTOF/MS, lettuce samples (200 mg) were ground into powder in liquid nitrogen, and extracted in 1 mL of methanol/water (80:20, v/v) for negative ion mode analysis^19^ or 1 mL of acidulated methanol/water solution (80:19.5:0.5 v/v/v mixture of methanol, water and 0.1 M HCl) for positive ion mode analysis. Samples (3 μL) were separated using an Acquity UPLC HSS T3 column (100 mm × 2.1 mm, i.d.,1.7 μm; Waters Corp., Milford, MA, USA) at 30 °C. The mobile phases were water containing 0.1% methanoic acid (A) and acetonitrile containing 0.1% methanoic acid (B), and the flow rate was 0.4 mL min^−1^. Gradient elution for negative mode was 0–3 min, 0% B; 3–3.1 min, 0–5% B; 3.1–6 min, 5–20% B; 6–11 min, 20–50% B; 11–15 min, 50–100% B; 15–17 min, 100% B, then initial conditions were maintained for 5 min to equilibrate the column. For positive mode, the conditions were: 0–1.5 min, 1% B; 1.5–5 min, 1–25% B; 5–8 min, 25–50% B; 8–13 min, 50–100% B; 13–15 min, 100% B; 15–15.5 min, 100–1% B; 15.5–17.5 min, 1% B, then initial conditions were maintained for 5 min.

For MS, capillary voltage was 1.5 kV (negative mode) or 1 kV (positive mode), source temperature was 115 °C, desolvation temperature was 500 °C, desolvation gas flow was 1000 L h^−1^, collision energy was 20–40 eV, the scan range was 50–1000 m/z, and spectra were acquired in positive and negative ion mode. MS scanning was performed using a High Definition MS^e^ (data independent acquisition type in ion mobility). QC samples were run at the beginning, middle and end of each batch.

#### Data pre-process

GC × GC-TOF-MS and UPLC-IMS-QTOF-MS/MS data were subjected to a series of processing procedures, including baseline correction, denoising, smoothing, time-window splitting, deconvolution, and peak alignment using LECO Chroma TOF (LECO Corp.) and Progenesis QI (Waters Corp.) software, respectively.

### Compound identification and pathway analysis

Metabolites separated by GC × GC-TOF-MS were identified using LECO Chroma TOF software by reference to online and local databases, including the NIST 2014 mass spectral database (Scientific Instrument Services, Inc. NJ, USA) and LECO/Fiehn Metabolite mass spectral library (Version 1.00, LECO Corp.). Fiehn retention index values were calculated using LECO Chroma TOF software with reference to the fatty acid methyl ester (C6-C24)^22^. Mass spectral matching was manually supervised with a match threshold of >650 (maximum 1000). Peak areas for each metabolite were based on selected quantification masses.

UPLC-IMS-QTOF-MS metabolites identification was performed in UNIFI Scientific Information System software (Waters Corp.) and based comparison of accurate mass, retention time, MS^2^ fragments and CCS values with online reference databases including Respect (http://spectra.psc.riken.jp/), Metlin (https://metlin.scripps.edu/), HMDB (http://www.hmdb.ca/), Lipidmap (http://www.lipidmaps.org/), in-house databases based on commercial standards and theoretical MS^2^ tags, and bibliographies. The CCS value acceptable error was <5%; with MS tolerance of 5 p.p.m., and MS/MS tolerance of <10 mDa, at least one major fragment was found.

### **Data analysis**

A three-dimensional data matrix, including the metabolite name (putatively identified by GC × GC-TOF-MS and UPLC-IMS-QTOF-MS), sample information (six biological repeats for each sample), and raw abundance (peak area for each putatively identified metabolite) was generated, uploaded to MetaboAnalyst 4.0 (http://www.metaboanalyst.ca/) analyzed according to the instructions provided. Raw data were subjected to three categories of normalization: normalization by median, log transformation, and auto data scaling. Next, R statistical environment and MetaboAnalyst 4.0 were used to perform univariate analysis (fold change analysis, *t*-tests, volcano plots), multivariate analysis (principal component analysis [PCA], partial least squares-discriminant analysis [PLS-DA]), K-means clustering analysis (Euclidean distances, Ward clustering algorithm) and random forest classification ([RF] with 500 trees, seven predictors). A correlation matrix containing all possible pair-wise Spearman’s rank correlations between putatively identified metabolites was generated to visualize the network correlations. Robust correlations between two metabolites was defined as a Spearman’s correlation coefficient (*p*) >0.75 and *P* < 0.01^23^. Networks were drawn using the Gephi platform (based on Java Virtual Machine, version 0.9.2, https://gephi.org/) with the Fruchterman Reingold algorithm. The metabolic pathway analysis was performed using online databases such as KEGG (http://www.kegg.jp/) and PlantCyc (http://www.plantcyc.org/). Metabolic pathways were visualized using ProcessOn (https://www.processon.com/).

## Results

### Morphological features

A total of 30 varieties of lettuce (six original from America, 12 original from Asia, 10 original from Europe and two of unknown origin) representing large phenotypic variations, such as leaf color, leaf shape, leaf shape of tip, type of undulation leaf margin, leaf texture, glossiness of leaf upper side, degree of undulation of leaf blade margin and head type (Supplemental Table S1 and Figure S1) were studied. For example, the leaf color of seven cultivars was amaranth or light amaranth, nine were yellow–green, six were green, five were light green, and three were dark green.

### Metabolite identification

GC × GC-TOF/MS and UPLC-IMS-QTOF/MS-based untargeted metabolomic approaches were performed to profile the metabolites present in 30 leaf and head lettuce cultivars. After pre-processing, 6782 features were extracted from the GC × GC-TOF/MS matrix by LECO Chroma TOF software (Supplemental Figure S2A), and 27,927 features (14,541 in negative mode and 13,386 in positive mode) were extracted from the UPLC-IMS-QTOF/MS data by Progenesis QI software (Supplemental Figure S2B). However, due to the complexity of structural isomers, the analytical platform selectivity, the chromatographic reproducibility between different platforms (or columns) and the lack of available databases with MS^2^ tags, metabolites identification in non-targeted metabolomics studies remains a remarkable and time-consuming challenge^14,15^.

The GC × GC-TOF/MS matrix was compared to the NIST 2014 mass spectral database and the LECO/Fiehn Metabolite mass spectral library. Using a match threshold >650 and Fiehn RI deviation <5% followed by manual supervision, a total of 76 compounds were identified, mainly small-polar compounds including amino acids, organic acids and carbohydrates (Supplemental Table S2**)**.

UPLC-IMS-QTOF/MS identified were compared with the Waters in-house lettuce database and the Metlin, ResPect, and Lipids databases, as well as the literature and commercial standards to identify the semi-polar or polar metabolites. A total of 95 compounds were putatively identified, including lipids, nucleotides, polyphenols (phenolic acids, flavonoids, anthocyanins), and terpenoids (Supplemental Table S3**)**.

The combination of 2D-GC × GC and TOF-MS enable the acquisition velocity up to 50 spectra s^−1^ to reconstruct chromatogram, which facilitates resolving analytes and enables the detection of chromatographic features up to thousands in a single analytical run. For instance, we extracted 1815 features in a repeat of sample s13K072 by setting noise criterion of 50 in LECO Chroma TOF software.

**Fig. 1:**
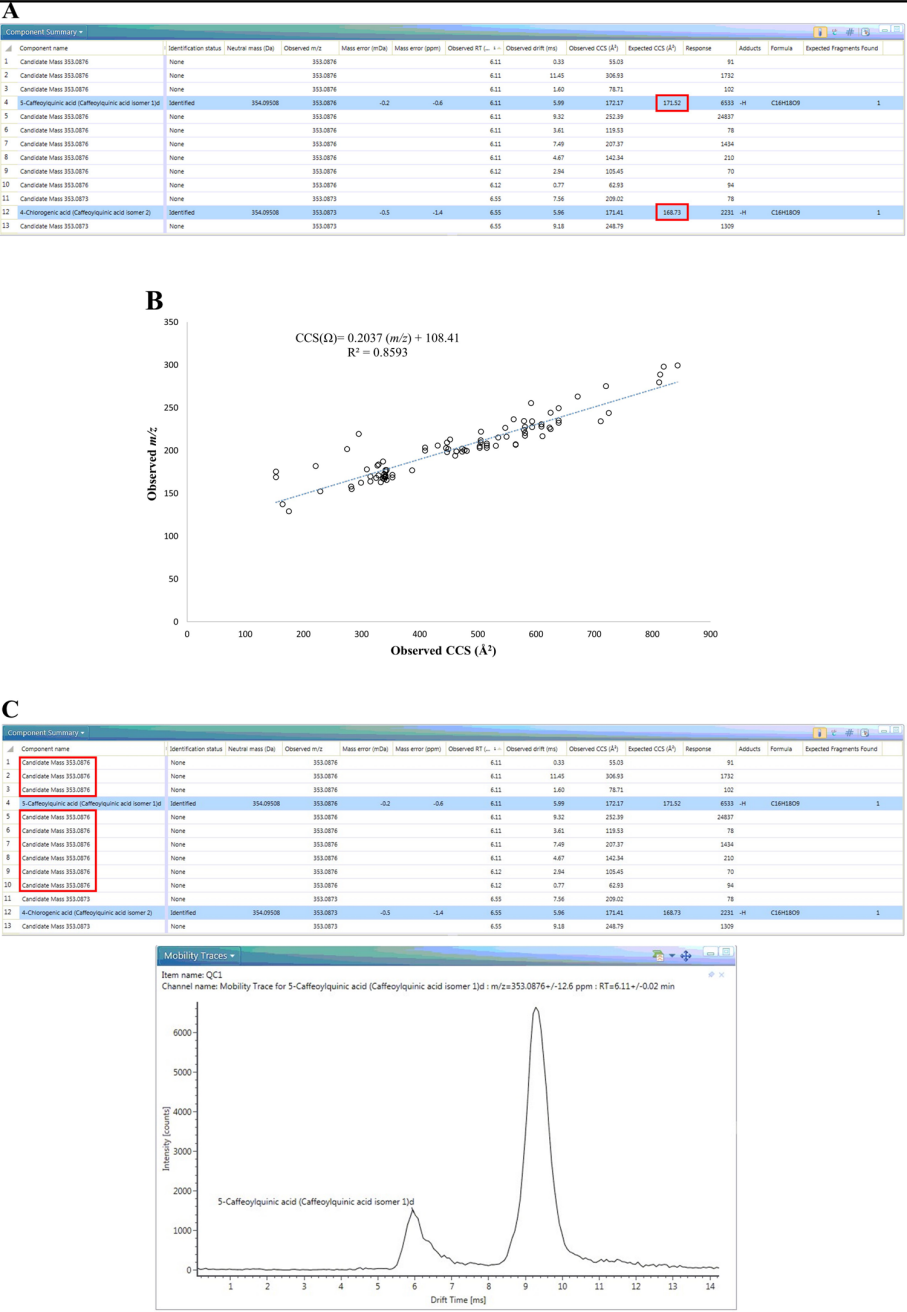
CCS offers great help for metabolites identification. **a** CCS value helps the identification of isomers; **b** the relationship between CCS value and *m/z* (*n* = 95), **c** CCS value helps screen false positive results

The coupling of traveling-wave ion-mobility technology to MS is a powerful tool both for metabolite separation and structural elucidation. The CCS values generated by IMS in this study help separate a number of isomers. For example, the candidates putatively identified as No. LC 110 and 116 (Supplemental Table S3), had the same *m/z*, MS^2^ fragments and neighboring retention times, but different CCS values (CV <2%), offering additional evidence supporting their identification as two isomers (Fig. 1a, feature 4 and 12). Moreover, a strong positive correlation was observed between the CCS values and respective molecular masses of the metabolites (*R*^2^ = 0.86, Fig. 1b). Similarly, previous analyses of peptides^24^, phenolics^25^, and lipids^26^ standard molecules with high *m/z* indicated that these metabolites might undergo greater collision than the smaller molecules, resulting in longer drift time in the buffer gas chamber. Therefore, using the CCS value, it is easy to eliminate these false positive results. For example, features 1–10 had the same *m/z* and observed retention time but different CCS values. Based on the discrepancy between the *m/z* and calculated CCS value of 180.33 Å^2^ (the CCS value of the standard was 171.52 Å^2^), we removed false identification results (Fig. 1c).

### Metabolic profiles of lettuce

The putatively identified 171 compounds contained 17 amino acids, 21 carbohydrates, 14 lipids, five nucleotides and derivatives, 39 organic acids, 59 polyphenols (phenolic acids, flavonoids, and anthocyanins), eight terpenoids and eight other metabolites. According to the proposed minimum metadata for metabolite identification^27^, 36 metabolites were identified as level 1 (identified compounds), 86 metabolites were level 2 (putatively annotated), and 49 metabolites were level 3 (putatively characterized compound classes).

**Fig. 2:**
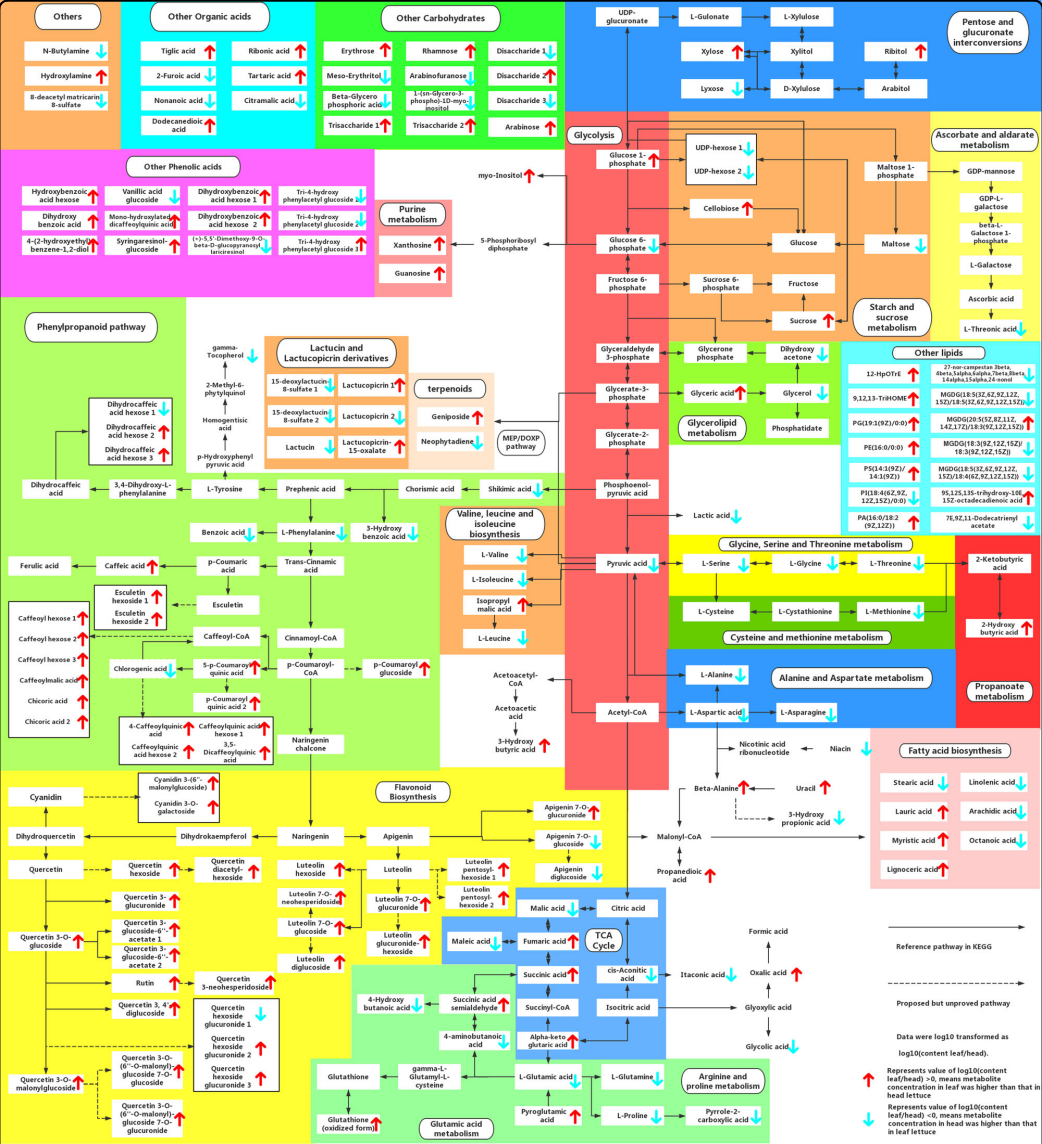
Metabolic pathways of lettuce. The 171 metabolites identified were mapped onto primary and secondary metabolism. The raw abundance of each metabolite was calculated by peak area. Then raw abundance was normalized by MetaboAnalyst, then mean of normalized peak area data were log10 transformed as log10 (content leaf/head). The upward-pointing red arrows represent the value of log10 (content leaf/head) >0, means higher levels of metabolites in leaf lettuces compared to head lettuces. While the value of log10(content leaf/head) <0 means lower levels of metabolites in leaf lettuces compared to head lettuces, and represented by downward-pointing blue arrows

We performed pathway analysis by comparing the metabolites with the KEGG and PlantCyc reference pathway. The most relevant pathways were alanine, aspartate, and glutamate metabolism; the citrate cycle; valine, leucine, and isoleucine biosynthesis; arginine and proline metabolism; glycine, serine, and threonine metabolism; glycolysis; fatty acid biosynthesis; glycerolipid metabolism; starch and sucrose metabolism; phenylpropanoid biosynthesis and flavonoid biosynthesis. The metabolites identified were mapped these onto metabolic pathways, which included both primary and secondary metabolism (Fig. 2).

### Specific metabolites in leaf and head type lettuces

To assess the metabolic differences between the leaf and head types of lettuce, we compared the relative contents of the 171 putatively identified metabolites (Fig. 2). The leaf types contained high levels of phosphatidyl lipids (e.g., PA, PE) and phenolic compounds, including hydroxybenzoic (e.g., dihydroxybenzoic acid), hydroxycinnamic (e.g., caffeoyl-hexose isomers), and glycosylated quercetin and luteolin. However, head type lettuces contained high concentrations of amino acids, long chain fatty acids (e.g., linolenic acid), most terpenoids (e.g., lactucin) and some monogalactosyl diglyceride (MGDG) lipids.

**Fig. 3:**
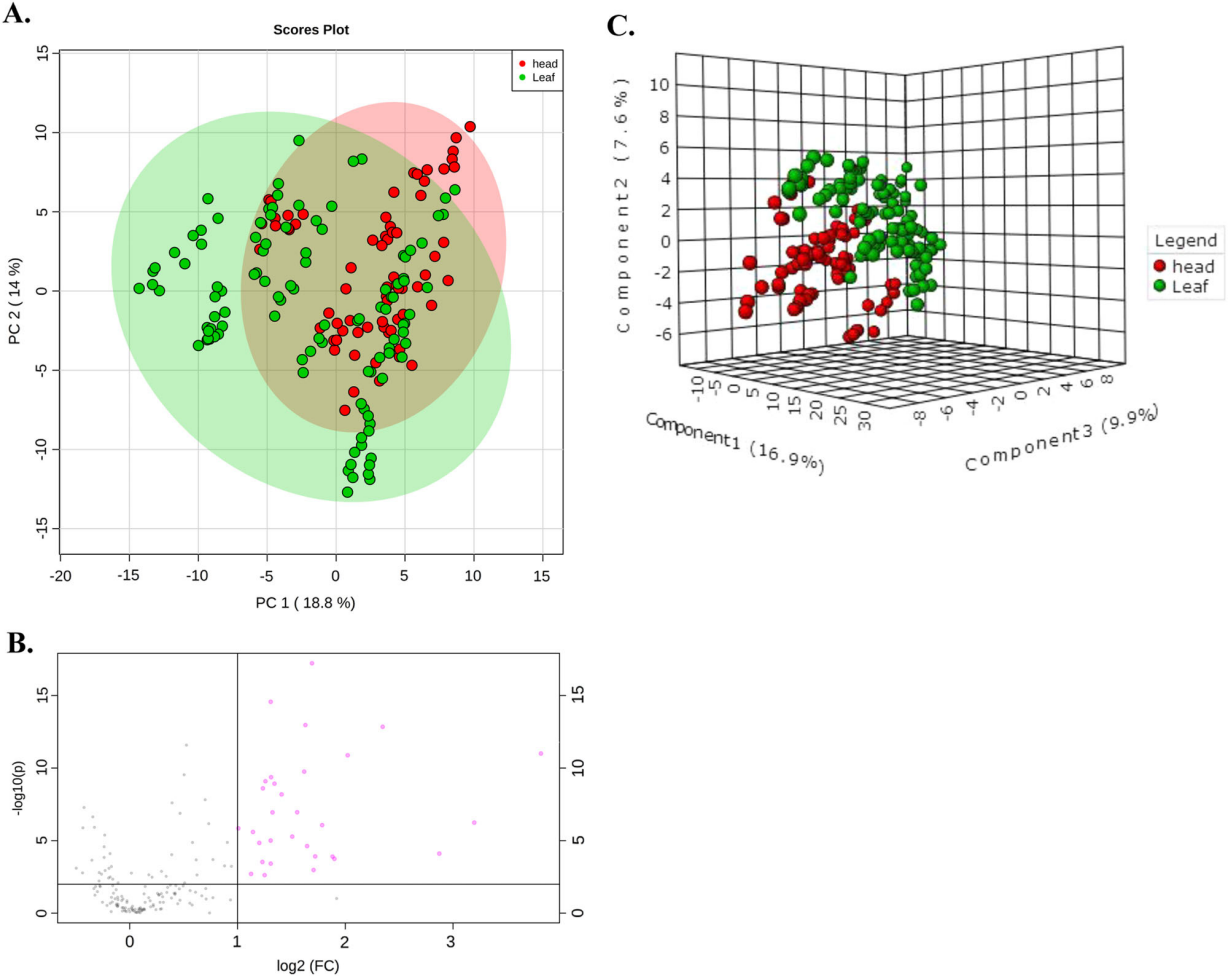
The data analysis of the metabolites in leaf and head lettuces. **a** PCA analysis, the green and red circle display 95% confidence regions of leaf and head groups; **b** Volcano plot analysis, features with a fold-change threshold of two and *t*-test *P* < 0.01 were included in the volcano plots. Red circles represent features above the threshold; **c** PLS-DA analysis

Next, we performed unsupervised principal component analysis (PCA) and *K*-means clustering analysis to assess the variations in the 171 metabolites detected across the 30 lettuce cultivars. The PCA and clustering analysis separated the leaf and head lettuce cultivars, although some of the samples overlapped (Fig. 3a and supplemental Figure S3), and revealed differences in the levels of metabolites present in leaf and head lettuces cultivated under the same growth conditions.

Additional methods, including volcano plots, PLS-DA and RF, were used to further explore the differences in metabolites between the leaf and head type lettuces. Metabolites with a fold change >2 and *P*-value (*t-*test) <0.01 were included in the volcano plot (Fig. 3b and Supplemental Table S4). The PLS-DA model based on first three components (accuracy = 0.94, *R*^2^ = 0.81, and Q^2 ^= 0.73) also revealed an apparent separation between the leaf and head types (Fig. 3c). The first three components explained 34.4% of the total variance. Then, we selected parameters of variable importance in projection (VIP) >1.2 for further screening (Supplemental Table S5). Moreover, RF was performed to group the leaf and head types and identify potential candidate metabolites that contribute to the differences between these cultivars. By setting 500 classification trees, RF separated the leaf and head types with an out-of-bag error value of 0, indicating the model was highly robust (Supplemental Figure S4). The 40 metabolites with highest mean decrease accuracy value were listed in the RF analysis (Supplemental Table S6).

Collectively, the three independent methods of metabolite analysis indicated 16 individual metabolites represent ‘candidates’ that can discriminate between leaf and head types of lettuce (Table 1). Leaf type lettuces contained higher concentrations of all 16 candidate compounds than the head types. The 16 candidate metabolites, including hydroxycinnamic acid, dihydroxybenzoic acid, glycosylated quercetin, luteolin, and one iridoid, are mainly involved in the phenylpropanoid, flavonoid and terpenoid pathways; these pathways are associated with plant developmental regulation, stress response and insect resistance^28–31^.

**Table 1:** Differential metabolites in leaf and head lettuce cultivars

No.	Candidate name	VIP score (component 1)	Fold change (leaf/head)	Mean decrease accuracy	*P*-value	FDR
LC_95	Caffeoylquinic acid hexose isomer 1	2.50	3.23	1.53E−02	8.12E−17	1.39E−14
LC_100	Caffeoylquinic acid hexose isomer 2	2.33	2.48	1.95E−02	7.24E−15	6.19E−13
LC_141	Quercetin 3-glucoside -6″-acetate (isomer 1)	2.21	3.09	8.37E−03	5.61E−14	3.20E−12
LC_144	Quercetin 3-glucoside -6″-acetate (isomer 2)	2.20	5.09	1.34E−02	1.15E−13	4.91E−12
LC_149	Quercetin diacetyl-hexoside	2.05	14.08	7.22E−03	9.79E−12	3.35E−10
LC_113	Quercetin 3-O-(6″-O-malonyl)-glucoside 7-O-glucoside	2.04	4.06	1.01E−02	2.20E−11	6.27E−10
LC_122	Luteolin di-glucoside	1.93	3.07	7.95E−03	1.51E−10	3.69E−09
LC_102	Dihydrocaffeic acid hexose isomer 2	1.90	2.48	1.03E−02	6.71E−10	1.43E−08
LC_90	Dihydroxybenzoic acid	1.87	2.39	1.32E−02	1.17E−09	2.22E−08
LC_112	Quercetin 3-O-(6″-O-malonyl)-glucoside 7-O-glucuronide	1.85	2.54	7.12E−03	1.87E−09	3.19E−08
LC_106	Dihydrocaffeic acid hexose isomer 3	1.82	2.36	5.11E−03	7.33E−09	1.14E−07
LC_120	Quercetin hexoside glucuronide isomer 3	1.77	2.66	6.91E−03	2.17E−08	2.85E−07
LC_135	Luteolin 7-glucuronide	1.63	2.51	4.10E−03	8.65E−08	7.79E−07
LC_103	Geniposide	1.55	9.17	1.10E−02	1.22E−06	9.92E−06
LC_97	Caffeoyl-hexose isomer 1	1.35	2.30	5.38E−03	6.22E−05	2.96E−04
LC_117	Luteolin glucuronide-hexoside	1.32	3.13	1.70E−02	3.02E−05	1.52E−04

**Fig. 4:**
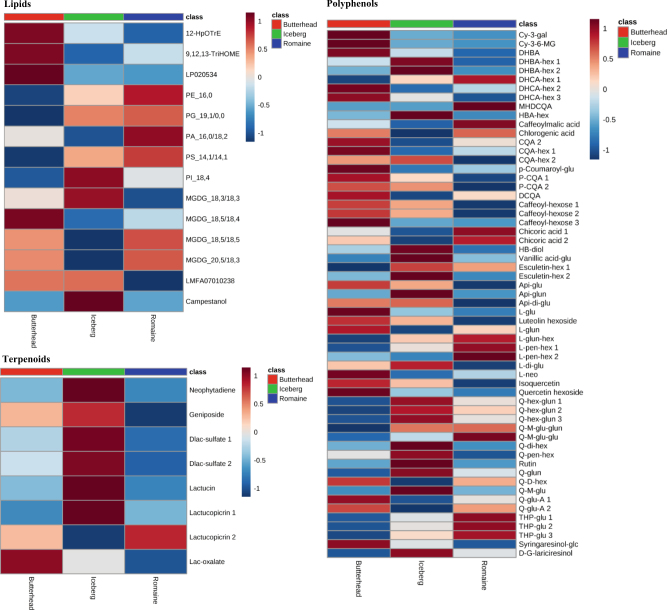
Heatmaps of the relative content of lipids, terpenoids, and polyphenols in head lettuces. For lipids; LP020534, 9S,12S,13S-trihydroxy-10E,15Z-octadecadienoic acid; PE_16, 0, PE(16:0/0:0); PG_19, 1/0, 0, PG(19:1(9Z)/0:0); PA_16, 0/18, 2, PA(16:0/18:2(9Z, 12Z)); PS_14, 1/14, 1, PS(14:1(9Z)/14:1(9Z)); PI_18, 4, PI(18:4(6Z, 9Z, 12Z, 15Z)/0:0); MGDG_18, 3/18, 3, MGDG(18:3(9Z, 12Z, 15Z)/18:3(9Z, 12Z, 15Z)); MGDG_18, 5/18, 4, MGDG (18:5(3Z, 6Z, 9Z, 12Z, 15Z)/18:4(6Z, 9Z, 12Z, 15Z)); MGDG_18, 5/18, 5, MGDG (18:5 (3Z, 6Z, 9Z, 12Z, 15Z) / 18:5 (3Z, 6Z, 9Z, 12Z, 15Z)); MGDG_20, 5/18, 3, MGDG(20:5 (5Z, 8Z, 11Z, 14Z, 17Z)/18:3(9Z, 12Z, 15Z)); LMFA07010238, 7E,9Z,11-Dodecatrienyl acetate; Campestanol, 27-nor-campestan-3beta, 4beta, 5alpha, 6alpha, 7beta, 8beta, 14alpha, 15alpha, 24-nonol; For terpenoids; Dlac-sulfate 1, 15-deoxylactucin-8-sulfate 1; Dlac-sulfate 2, 15-deoxylactucin-8-sulfate 2; Lactucopicrin 1, Lactucopicrin isomer 1; Lactucopicrin 2, Lactucopicrin isomer 2; Lac-oxalate, Lactucopicrin -15-oxalate. For polyphenols; Cy-3-gal, Cyanidin 3-O-galactoside; Cy-3-6-MG, Cyanidin 3-(6″-malonylglucoside); DHBA, Dihydroxybenzoic acid; DHBA-hex 1; Dihydroxybenzoic acid hexose isomer 1; DHBA-hex 2, Dihydroxybenzoic acid hexose isomer 2; DHCA-hex 1, Dihydrocaffeic acid hexose isomer 1; DHCA-hex 2, Dihydrocaffeic acid hexose isomer 2; DHCA-hex 3, Dihydrocaffeic acid hexose isomer 3; MHDCQA, Mono-hydroxylated dicaffeoylquinic acid; HBA-hex, Hydroxybenzoic acid hexose; Chlorogenic acid, 5-Caffeoylquinic acid (Caffeoylquinic acid isomer 1); CQA 2, 4-Caffeoylquinic acid (Caffeoylquinic acid isomer 2); CQA-hex 1, Caffeoylquinic acid hexose isomer 1; CQA-hex 2, Caffeoylquinic acid hexose isomer 2; P-CQA 1, 5-p-coumaroylquinic acid (p-coumaroylquinic acid isomer 1); P-CQA 2, p-coumaroylquinic acid isomer 2; DCQA, 3,5-Dicaffeoylquinic acid; Caffeoyl-hexose 1, Caffeoyl-hexose isomer 1; Caffeoyl-hexose 2, Caffeoyl-hexose isomer 2; Caffeoyl-hexose 3, Caffeoyl-hexose isomer 3; Chicoric acid 1, Chicoric acid; Chicoric acid 2, Chicoric acid (isomer 2); HB-diol, 4-(2-hydroxyethyl)benzene-1,2-diol; Vanillic acid-glu, Vanillic acid glucoside; Esculetin-hex 1, Esculetin hexoside isomer 1; Esculetin-hex 2, Esculetin hexoside isomer 2; Api-glu, Apigenin 7-O-glucoside; Api-glun, Apigenin 7-O-glucuronide; Api-di-glu, Apigenin di-glucoside; l-glu, Luteolin 7-glucoside; Luteolin hexoside, Luteolin hexoside (isomer 2); l-glun, Luteolin 7-glucuronide; l-glun-hex, Luteolin glucuronide-hexoside; l-pen-hex 1, Luteolin pentosyl-hexoside isomer 1; l-pen-hex 2, Luteolin pentosyl-hexoside isomer 2; l-di-glu, luteolin di-glucoside; l-neo, Luteolin 7-neohesperidoside; Isoquercetin, Quercetin 3-glucoside; Q-hex-glun 1, Quercetin hexoside glucuronide isomer 1; Q-hex-glun 2, Quercetin hexoside glucuronide isomer 2; Q-hex-glun 3, Quercetin hexoside glucuronide isomer 3; Q-M-glu-glun, Quercetin 3-O-(6″-O-malonyl)-glucoside 7-O-glucronide; Q-M-glu-glu, Quercetin 3-O-(6″-O-malonyl)-glucoside 7-O-glucoside; Q-di-hex, Quercetin 3, 4′-di-glucoside; Q-pen-hex, Quercetin 3-neohesperidoside; Rutin, Quercetin 3-rutinoside (rutin); Q-glun, Quercetin 3-glucuronide; Q-D-hex, Quercetin diacetyl-hexoside; Q-M-glu, Quercetin 3-(6″-malonylglucoside); Q-glu-A 1, Quercetin 3-glucoside -6″-acetate (isomer 1); Q-glu-A 2, Quercetin 3-glucoside -6″-acetate (isomer 2); THP-glu 1, Tri-4-hydroxyphenylacetyl glucoside isomer 1; THP-glu 2, Tri-4-hydroxyphenylacetyl glucoside isomer 2; THP-glu 3, Tri-4-hydroxyphenylacetyl glucoside isomer 3; Syringaresinol-glc, Syringaresinol-glucoside; D-G-lariciresinol, (+)-5,5′-Dimethoxy-9-O-betaD-glucopyranosyl lariciresinol

We also compared the concentrations of the metabolites between different head lettuces, including romaine, iceberg, and butterhead (Fig. 4 and Supplemental Figure S5). Romaine lettuce contained the highest levels of some acidic amino acids (Asp and Glu), Met and Gln, pentoses (arabinose, xylose, and lyxose), phospholipids, including phosphatidylglycerol (PG), phosphatidic acid (PA) and phosphatidylserine (PS), and some monogalactosyl diglycerides (MGDGs); nucleic acid derivatives, some organic acids related to the citrate cycle (α-ketoglutaric acid and *cis*-aconitic acid), niacin and phenolic compounds (tri-4-hydroxyphenylacetyl glucoside isomers); chlorogenic acid, chicoric acid and some acylated di-glycosides of quercetin and luteolin. Compared to romaine and butterhead lettuces, iceberg lettuce contained higher levels of some non-polar amino acids, such as Ala, Val, Leu, Ile, Gly, and Phe, disaccharides and trisaccharides, some lipids including sterol and phosphatidylinositol (PI), hydroxybutyric acid isomers, benzoic acid, 2-furoic acid; succinate semialdehyde, malic acid, shikimic acid, some fatty acids (octanoic, stearic, linolenic, and lignoceric acid), most terpenes, dihydroxybenzoic acid hexose isomers, esculetin hexoside isomers, and some glycosides of quercetin and apigenin. Among the head type lettuces, butterhead lettuces had the highest levels of proline, 4-aminobutyric acid, myo-inositol, phenolics, including dihydroxybenzoic acid, dihydrocaffeic acid hexose isomers, glycosides of luteolin and quercetin, and apigenin; all of these metabolites have been associated with osmotic resistance^32–35^. Butterhead lettuces also had the highest levels of octadecadienoic acids, pyruvic acid, lactic acid, oxalic acid, maleic acid, succinic acid, fumaric acid, and caffeic acid.

### Network-based analysis reveals metabolite correlations between leaf and head lettuces cultivars

**Fig. 5:**
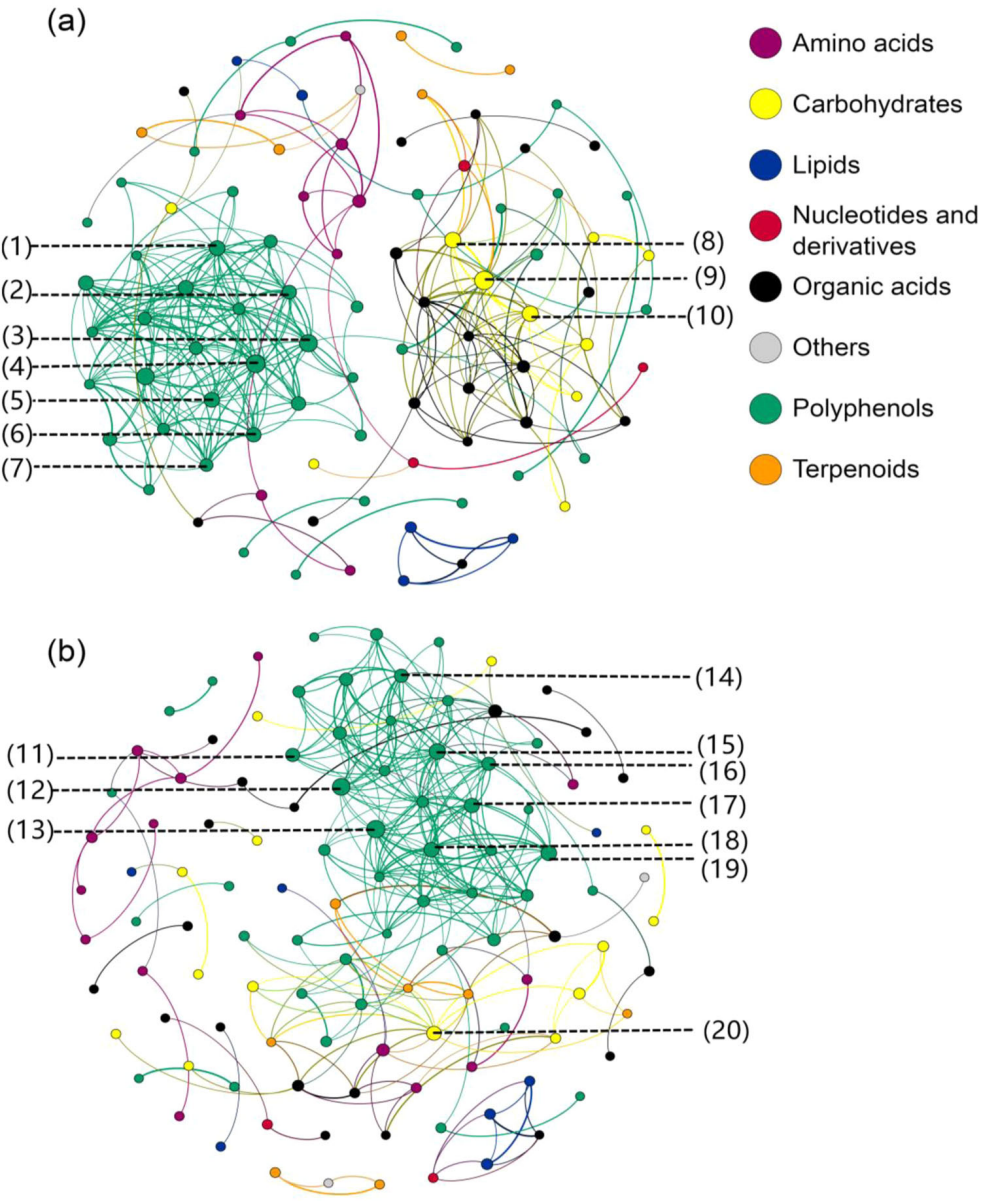
Network analysis of the metabolites in leaf and head lettuces. **a** Leaf lettuce network; **b** head lettuce network; (1) Luteolin di-glucoside; (2) Quercetin hexoside glucuronide isomer 2; (3) Quercetin 3-O-(6″-O-malonyl)-glucoside 7-O-glucoside; (4) Quercetin 3, 4′-di-glucoside; (5) Quercetin 3-O-(6″-O-malonyl)-glucoside 7-O-glucronide; (6) Caffeoylmalic acid; (7) Quercetin hexoside glucuronide isomer 3; (8) Xylose; (9) Arabinose; (10) Ribitol; (11) Esculetin hexoside isomer 1; (12) Caffeoylquinic acid hexose isomer 2; (13) 5-Caffeoylquinic acid (Caffeoylquinic acid isomer 1); (14) Quercetin hexoside glucuronide isomer 1; (15) Quercetin 3-O-(6″-O-malonyl)-glucoside 7-O-glucoside; (16) Luteolin 7-neohesperidoside; (17) Caffeoylmalic acid; (18) Chicoric acid; (19) 4-Caffeoylquinic acid (Caffeoylquinic acid isomer 2); (20) Lyxose

Network-based analysis was used to assess metabolite correlations and interactions between leaf and head lettuce cultivars and reveal potential regulatory elements (Fig. 5a, b). The network of leaf type lettuce metabolite contained 94 nodes and 287 edges, with node connectivity of 2.5, the average path length of 1.7 edges and network diameter of 5 edges. The network of head type lettuce metabolite was denser, with 113 nodes and 282 edges, node connectivity of 2.1, the average path length of 2.1 and network diameter of 7 (Supplemental Table S7). Based on modularity, the leaf type metabolite network contained four major modules, while the head type metabolite network contained three major connected components (Supplemental Figure S6A, S6B and Table S7).

Polyphenols represented the largest module in both the leaf (27.66% of total network connections) and head (21.24% of total network connections) lettuce metabolite networks, representing 25 nodes with 163 edges in leaf types and 20 nodes with 121 edges in head types (Supplemental Figure S6A, S6B). We then identified the most well-connected nodes in the networks. In the leaf type metabolite network, the top 10 nodes were three carbohydrates (arabinose, ribitol, xylose), six quercetin and luteolin derivatives (quercetin 3, 4′-di-glucoside, quercetin 3-*O*-(6″-*O*-malonyl)-glucoside 7-*O*-glucoside, quercetin hexoside glucuronide isomer 2 and 3, luteolin di-glucoside, quercetin 3-*O*-(6″-*O*-malonyl)-glucoside 7-*O*-glucuronide), and one phenolic acid (caffeoylmalic acid). In contrast, six phenolic acid derivatives (chlorogenic acid, caffeoylquinic acid hexose isomer 2, caffeoylquinic acid isomer 2, caffeoylmalic acid, chicoric acid, esculetin hexoside isomer 1), three quercetin and luteolin derivatives (quercetin 3-*O*-(6″-*O*-malonyl)-glucoside 7-*O*-glucoside, luteolin 7-neohesperidoside, quercetin hexoside glucuronide isomer 1), and one carbohydrate (lyxose) were the most well-connected 10 nodes in the head type lettuce metabolic network.

## Discussion

### GC × GC-TOF/MS combined with UPLC-IMS-QTOF/MS is a powerful tool for profiling lettuce metabolites

To the best of our knowledge, a large-scale untargeted metabolomic study based on GC × GC-TOF/MS combined with UPLC-IMS-QTOF/MS has not been reported for any plant species. Construction of our lettuce metabolite library based on this combination of analytical techniques, together with our non-targeted metabolomic profiling method, provided precise, comprehensive data that enabled simultaneous detection and quantification of both primary and secondary metabolites. This approach could also be used to study metabolites in other plant species.

GC × GC-TOF/MS is an effective technique for initiation of small-polar compounds that offers a higher peak capacity, better separation, and easier metabolite identification compared to 1D-GC^36^. GC × GC-TOF/MS enabled detection of more than 1800 features in a single sample in this study, which was threefold higher than the number of features detected by 1D-GC/MS using the same noise criterion (>50) and same software (LECO Chroma TOF) in our previous study^19^.

UPLC-IMS-QTOF/MS separates ions based on their size, shape, ionic interactions with the buffer gas and charge state in the gas chamber, and generates a CCS value based on ion drift time, which can be used to refine the identification of complex metabolites, particularly isomeric compounds^26^. Moreover, IMS-based MS significantly improved the analysis rate of high throughput sample analysis and significantly decreased the requirement for chromatographic separation before MS analysis^17^. In this study, by using the selectivity of IMS, precursors and MS fragments of detected metabolites was time-aligned in a 17 min analysis. The chromatographic separation time in this study decreased 11 min^7^ or 20 min^12^ compared to previous studies performed with the negative mode in Waters UPLC-QTOF-MS platforms.

### Biological relevance of the metabolic differences between lettuce types

Lettuce cultivation has a long history. Humans began to select lettuce traits at least 4500 years ago, and these processes have dramatically changed the molecular phenotypes and morphological features (e.g., resistance to bolting, preference for shorter and broader leaf shape, a decrease in latex content, and hearting characteristics)^37,38^. To investigate the relationship between morphological features and metabolites, we calculated metabolites PCA score of different lettuce accessions based on their different phenotypical features and original sources. No clear separation in PCA score plots was observed based on the parameters of leaf color, leaf shape, leaf texture, and original source (Supplemental Figure S7A, S7B, S7C and S7D). However, we found a division of two groups related to original lettuce sources (Supplemental Figure S7E). Plots distribution suggested that European lettuce cultivars were the main components of group one; lettuces from the Asia were the major factors of Group two, and American lettuces contributed to both group one and two.

Our non-targeted metabolomic approach based on UPLC-IMS-QTOF/MS combined with GC × GC-TOF/MS revealed 16 candidate metabolites, including six phenolic acid derivatives, nine glycosylated flavonoids, and one iridoid, could significantly separate the leaf and head types of lettuce. Surprisingly, almost all the candidate features involved in polyphenol metabolism accumulated at higher levels in leaf lettuce than head lettuce, suggesting a vital role for phenolic compounds in discrimination of head and leaf lettuce types. Phenolic acids (including hydroxycinnamic and dihydroxybenzoic derivatives) serve as intermediates in lignin biosynthesis and are involved in the lignification process^39^. Unlike other phenolic acids, caffeoylquinic acid derivatives (e.g., caffeoylquinic acid hexose isomer 1 and 2 identified in this study) are located in chlorenchyma cells^39^ that potentially protect chloroplasts from ultraviolet radiation damage^40^. Glycosylated quercetin and luteolin are more powerful reactive oxygen species scavengers than other flavonoids^41^; their accumulation has been associated with plant resistance of UV radiation. Accumulation of polyphenols during biosynthesis may be related to environmental adaptation (e.g., to drought, varying temperatures, ultraviolet radiation) or pest and disease resistance, and polyphenols serve as important factors in plant development^42^. Geniposide, a type of iridoid, was first extracted from jasmine and is one of the most widely distributed secondary metabolites in higher plants. Iridoids play significant roles in plant defense against herbivores and as intermediates linking the biosynthesis of terpenes and alkaloids^30,31^. Thus, the 16 differential candidates identified suggest the significant metabolomic variations between the leaf and head types of lettuce are related to secondary metabolism, particular resistance to biotic and abiotic factors. The leaf type lettuces contained higher concentrations of these compounds (including six phenolic acid derivatives, nine glycosylated flavonoids, and one iridoid), possibly due to their larger area of open/exposed leaf compared to head type lettuces. Even though leaf lettuces were first cultivated in Greece and Rome^37^, modern leaf lettuces may be the result of recent human selection with the aim of breeding higher concentrations of health-promoting compounds (e.g., antioxidants) due to increasing public demand for a healthy diet. Nevertheless, leaf type lettuces may have naturally evolved higher concentrations of secondary metabolites than head types as a mechanism of resistance to biotic and abiotic stresses.

Interestingly, products of sesquiterpene lactones (e.g., lactucin and lactucopicrin derivatives) constitute one of the primary mechanisms of protection against microbes (such as fungi, bacteria, and viruses) in the *Asteraceae* family^43^ as they disrupt the microbe phospholipid membrane^44^. These metabolites were accumulated at high levels in iceberg lettuce, indicating the existence of another strategy of plant resistance to environmental stress in lettuce, particularly in cultivars with low concentrations of flavonoids including quercetin and luteolin derivatives.

### Network analysis reveals metabolite diversity between leaf and head lettuces

Network analysis is a comprehensive approach that can be used to explore biochemical processes and their regulation based on metabolic differences^45^. We used network analysis to screen potential metabolic indicators and predict metabolic strategies^46^. Identifying the components with the main hubs (most-connected nodes) is a crucial prority^47^ due to the “central” role of these highly connected components in the middle of the network. Network analysis revealed that the tightly inter-regulated polyphenol module acts as a backbone; polyphenols was the most well-connected module in both the leaf and head types. Moreover, the most well-connected nodes in the condensed networks were also mainly involved in polyphenol biosynthesis. Polyphenols have been shown to contribute to higher plant development and plant/environment interactions, particularly anti-ultraviolet radiation and lignification^42^. However, the leaf and head types may have evolved different polyphenol metabolic strategies to adapt to the environment during evolution or human selection. Glycosylated flavonoids, particularly luteolin, and quercetin derivatives, represented a large number of hubs in the leaf type network, indicating a vital role for flavonoids in plant resistance, while the analysis indicated head type lettuces might employ phenolic acids (e.g., caffeoylquinic acids derivatives) for plant adaption and lignification. Furthermore, network analysis suggested pentose derivatives may play potentially critical functional roles in lettuce. Pentose derivatives are mainly associated with the pentose phosphate pathway, which links primary and secondary metabolism and generates numerous intermediates and precursors, such as fatty acids, flavonoids, lignins, and nucleotides for many downstream pathways, including nucleic acid biosynthesis.

By combining several types of metabolic analyses, we found that secondary metabolites, mainly polyphenols and flavonoids, play a significant role in differentiating the two types of lettuce. Further research should focus on exploring the roles of the 16 candidate differential metabolites in plant resistance in different types of lettuce (e.g., flavonoids in leaf types and sesquiterpene lactones in iceberg lettuce) and how these metabolites have adapted to exert the same functions via different metabolic strategies during evolutionary selection. A recent study has reported that dozens of genes are potentially associated with flavonoid biosynthesis in lettuce and that some of them still have an unknown function^3^. Also, the integrative analysis of metabolomic data with other ‘omics’ approaches (e.g., transcriptomics^3^ or genomics^2^) are the useful way to further elucidate gene functions and explain the evolutionary process of lettuce during natural and human selection. These efforts could be a great help to reveal the vital roles of different metabolites related to important agronomy traits, such as stress tolerance, disease resistance, and nutrition quality in different lettuce types. Moreover, combining association mapping techniques (e.g., quantitative trait locus analysis and genome-wide association studies) with metabolomic analyses may help to more precisely assess the contribution of genetic factors to metabolic variation and the contribution of metabolic variation to complex traits in different lettuce cultivars.

## Conclusion

Comparison with online databases, the published literature and standards enabled putative identification of 171 metabolites using GC × GC-TOF/MS and UPLC-IMS-QTOF/MS in 30 lettuce cultivars representing large genetic diversity. The lettuce metabolite library and metabolomic profiling methodology described in this study could be used to further characterize metabolites in lettuce or other plants. Sixteen metabolites were found to be significantly different between the leaf and head types of lettuce; these candidates were secondary metabolites and included phenolic acid derivatives, glycosylated flavonoids, and one iridoid. Network analysis revealed that the different types of lettuce have distinct metabolic strategies regarding both metabolite abundance and the corresponding associated metabolic networks. These findings provide important insights into metabolic adaptations in lettuce in response to nature and human selection, and pave the way for further metabolic studies to potentially improve lettuce quality, yield, and nutritional properties.

## Electronic supplementary material

Supplemental Tables

Supplemental Figures
