# Supplementary material for: A novel integrated non-targeted metabolomic analysis reveals significant metabolite variations between different lettuce (Lactuca sativa. L) varieties
Source: Hortic Res. 2018 Jun 25;5:33. doi: 10.1038/s41438-018-0050-1 (PMC6015802; doi:10.1038/s41438-018-0050-1)
Supplement: Supplementary file 1 — Supplemental Tables [file 41438_2018_50_MOESM1_ESM.pdf]

Supplemental Table S1 Genotypes and major morphological features of the lettuce cultivars used in this study

Sample no.	Type	Original source	GRC No. or trade name	Leaf color	Leaf shape	Leaf tip shape	Type of leaf margin undulation	Leaf texture	Glossiness of leaf upper side	Degree of undulation of leaf blade margin
s13k014	Head (R)	California, USA	PI_536728	Light green	Spatulate	Circular	Crenate	Smooth	Glossy	None
s13k035#*	Leaf	Netherlands	PI_342476-2	Yellow-green	Obovate	Circular	Crenate	Slightly curled	Glossy	None
s13k037	Head (I)	Macedonia	PI_358043-1	Green	Obovate	Circular	Entire	Slightly curled	Glossy	None
s13k044	Leaf	Netherlands	PI_342502-1	Yellow-green	Long elliptic	Obtuse	Entire	Slightly curled	Glossy	None
s13k057	Leaf	Turkey	PI_164937	Yellow-green	Pandurate	Obtuse	Crenulate	Rugous	Glossy	None
s13k066	Leaf	New York, USA	PI_536700	Yellow-green	Oval	Circular	Crenate	Rugous	Glossy	None
s13k068	Leaf	India	PI_271476-1	Amaranth	Pandurate	Obtuse	Crenate	Rugous	Glossy	None
s13k072	Leaf	USA	PI_601060	Amaranth	Spatulate	Circular	Double serrate	Rugous	Glossy	None
14k374	Leaf	Washington, USA	PI_617958-3	Amaranth	Obovate	Circular	Crenate	Slightly curled	Glossy	None
14k313	Head (R)	Greece	PI_491213*	Dark green	Long elliptic	Obtuse	Entire	Slightly curled	Glossy	None
14k320*	Head (R)	Netherlands	PI_342541-4	Green	Spatulate	Circular	Entire	Slightly curled	Glossy	None
s14k331	Leaf	China	W6_35654	Light green	Long elliptic	Acuminate	Entire	Smooth	Glossy	None
s14k332#*	Leaf	Pennsylvania, USA	PI_536777	Light green	Long elliptic	Obtuse	Entire	Smooth	Glossy	None
14k333#*	Leaf	Beijing, China	PI_391601-1	Light green	Pandurate	Obtuse	Entire	Slightly curled	Glossy	None
14k339	Leaf	California, USA	W6_29817-1	Amaranth	Lanceolate	Acute	Irregular crenate	Slightly curled	Glossy	Deep
14k342	Leaf	Netherlands	PI_342474-1	Yellow-green	Long elliptic	Circular	Entire	Slightly curled	Glossy	None
14k360	Head (R)	Unknown	PI_140394	Green	Long elliptic	Obtuse	Entire	Smooth	Glossy	None
s15k059	Head (B)	Netherlands	PI_342556-4	Light amaranth	Oval	Circular	Entire	Slightly curled	Lustreless	None
s15k104*	Head (B)	Netherlands	PI_342481-1	Green	Oblate	Circular	Entire	Slightly curled	Lustreless	None
s15k118	Leaf	China	Hongzhou	Amaranth	Oblate	Obtuse	Crenulate	Rugous	Lustreless	Slight
15k119	Leaf	China	Yanzhi	Amaranth	Long elliptic	Obtuse	Crenate	Curled	Lustreless	None

15k121	Leaf	Viet Nam	VI046054	Yellow-green	Long elliptic	Acuminate	Entire	Slightly curled	Lustreless	None
s15k153	Head (B)	China	Flandria RZ	Green	Suborbicular	Circular	Entire	Smooth	Glossy	None
s15k157*	Leaf	Unknown	PI_178923-1	Yellow-green	Obovate	Circular	Crenulate	Curled	Glossy	Slight
s15k178*	Head (R)	Turkey	PI169495	Dark green	Long elliptic	Obtuse	Entire	Smooth	Lustreless	None
s15k203	Head (I)	China	Huanghou	Green	Suborbicular	Obtuse	Crenate	Slightly curled	Glossy	None
s15k204	Leaf	China	Luoshalv	Yellow-green	Suborbicular	Obtuse	Double serrate	Curled	Glossy	Slight
s15k218	Head (R)	China	Aoluo	Dark green	Pandurate	Obtuse	Entire	Smooth	Glossy	None
s15k225	Head (I)	China	Baoyulai	Light green	Suborbicular	Circular	Crenate	Rugous	Glossy	None
Bilvyi	Leaf	China	Bilvyi	Yellow-green	Obovate	Circular	Crenate	Slightly curled	Glossy	None

Note: Head (R), a romaine cultivar of head lettuce type; Head (I), an iceberg cultivar of head lettuce type; Head (B), a butterhead cultivar of head lettuce type

# This cultivar in field was observed different phenotype compared with the description on the original website. We changed the classification based on our observation.

* This cultivar was also used in a RNA-seq study conducted by Zhang et al., 2017.

Supplemental Table S2. Lettuce metabolites putatively identified by GC×GC-TOF/MS

No.	1st Dimension Time (min)	2nd Dimension Time (s)	*m/z* for quantification	Probable component	Similarity	Calculated Retention Index	Fiehn Retention Index	CV(%)	Level of identification
GC_1	8.63	2.14	157	Tiglic acid	773	206167	n.a	n.a	2
GC_2	9.30	2.42	174	Pyruvic acid	875	213962	210436	1.7	1
GC_3	9.56	2.08	117	Lactic acid	945	219607	216758	1.3	1
GC_4	9.96	2.14	147	Glycolic acid	888	232532	225852	3.0	2
GC_5	10.63	1.84	44	L-alanine	898	245617	244218	0.6	1
GC_6	10.63	1.96	174	N-butylamine	807	245656	n.a	n.a	2
GC_7	11.10	2.00	146	Hydroxylamine	901	254897	254023	0.3	2
GC_8	11.23	2.14	131	2-Hydroxybutyric acid	889	257580	n.a	n.a	2
GC_9	11.63	2.80	125	2-Furoic acid	807	265707	264112	0.6	2
GC_10	11.63	2.40	147	Oxalic acid	704	265576	260477	2.0	1
GC_11	11.70	2.24	66	3-Hydroxypropionic acid	843	266841	269050	-0.8	2
GC_12	12.16	2.18	147	3-Hydroxybutyric acid	883	276049	278679	-0.9	2
GC_13	12.90	2.62	89	Succinate semialdehyde	688	290696	294326	-1.2	2
GC_14	13.43	2.46	147	Propanedioic acid	881	301189	n.a	n.a	2
GC_15	13.76	2.04	144	L-valine	886	312269	314036	-0.5	1
GC_16	14.36	2.30	147	4-Hydroxybutanoic acid	900	319593	n.a	n.a	2
GC_17	14.76	2.36	70	Dihydroxyacetone	659	327522	333585	-1.8	2
GC_18	14.96	2.84	105	Benzoic acid	858	336912	339866	-0.8	1
GC_19	15.30	2.36	117	Octanoic acid	940	338069	n.a	n.a	1
GC_20	15.43	2.12	103	Glycerol	931	340626	345180	-1.3	2

GC_21	15.43	2.24	158	L-leucine	859	345655	346389	-0.2	1
GC_22	16.10	2.12	158	L-isoleucine	874	353809	359232	-1.5	1
GC_23	16.30	2.44	142	L-proline	848	363727	363983	-0.1	1
GC_24	16.43	2.78	147	Maleic acid	921	360618	365916	-1.4	2
GC_25	16.50	2.98	180	Niacin	888	362002	354525	2.1	1
GC_26	16.56	2.26	174	Glycine	892	367542	368260	-0.2	1
GC_27	16.76	2.62	55	Succinic acid	879	371517	371179	0.1	2
GC_28	17.23	2.28	103	D-glyceric acid	860	376273	377282	-0.3	2
GC_29	17.56	2.80	99	Uracil	850	382804	385872	-0.8	2
GC_30	17.63	2.64	147	Itaconic acid	874	383925	387056	-0.8	2
GC_31	17.83	2.34	45	Fumaric acid	898	390833	390675	0.0	1
GC_32	17.96	2.70	240	Pyrrole-2-carboxylic acid	776	389777	394475	-1.2	2
GC_33	18.10	2.16	204	L-serine	831	391954	395017	-0.8	1
GC_34	18.30	2.38	117	Nonanoic acid	853	395519	n.a	n.a	2
GC_35	18.90	2.24	117	Threonine	914	405981	410252	-1.0	1
GC_36	20.23	2.34	201	Erythrose	802	429350	435855	-1.5	2
GC_37	20.23	2.72	160	L-Aspartic acid	875	429461	432795	-0.8	2
GC_38	20.30	2.28	174	Beta-Alanine	866	430499	434448	-0.9	2
GC_39	21.56	2.38	247	Citramalic acid	841	452701	456194	-0.8	2
GC_40	21.97	2.46	55	Malic acid	924	459726	462908	-0.7	1
GC_41	22.56	2.08	147	Meso-Erythritol	923	470118	n.a	n.a	2
GC_42	22.63	2.94	100	Asparagine	824	471536	476536	-1.0	2
GC_43	23.03	2.66	176	L-methionine	828	478457	482597	-0.9	2
GC_44	23.16	3.76	84	Pyroglutamic acid	908	481111	485159	-0.8	1
GC_45	23.36	2.38	174	4-Aminobutanoic acid	887	484210	n.a	n.a	2
GC_46	23.56	2.24	147	L-Threonic acid	901	487645	497167	-1.9	2

GC_47	23.76	3.16	155	Glutamine	772	491207	491841	-0.1	2
GC_48	24.43	2.76	267	3-Hydroxybenzoic acid	796	502126	507006	-1.0	2
GC_49	24.63	2.94	198	Alpha-ketoglutaric acid	758	505484	507334	-0.4	2
GC_50	25.30	2.16	217	Arabinofuranose	798	516298	n.a	n.a	2
GC_51	25.83	2.44	128	L-Glutamic acid	820	525198	528609	-0.6	2
GC_52	26.23	2.42	147	Tartaric acid	899	531810	534818	-0.6	2
GC_53	26.50	2.28	103	Arabinose	888	536183	n.a	n.a	2
GC_54	26.76	2.28	103	Xylose	921	540595	542483	-0.3	2
GC_55	26.90	2.44	117	Lauric acid	876	542844	547162	-0.8	1
GC_56	27.63	2.22	103	Lyxose	797	554916	545540	1.7	2
GC_57	28.50	2.08	217	Ribitol	891	569214	576302	-1.2	2
GC_58	28.70	2.24	117	Rhamnose	864	572567	n.a	n.a	2
GC_59	29.36	2.60	229	cis-Aconitic acid	789	583461	587501	-0.7	2
GC_60	29.70	2.22	103	Ribonic acid	842	587694	n.a	n.a	2
GC_61	29.83	2.34	217	Glucose-1-phosphate	689	589445	594823	-0.9	2
GC_62	30.90	2.36	204	Shikimic acid	843	603255	n.a	n.a	2
GC_63	31.90	2.44	82	Neophytadiene	913	616215	n.a	n.a	2
GC_64	32.10	2.48	117	Myristic acid	853	618812	635876	-2.7	1
GC_65	34.83	2.12	219	Caffeic acid	817	654111	688718	-5.0	2
GC_66	36.43	1.60	305	Myo-inositol	828	720856	729867	-1.2	2
GC_67	36.96	1.70	143	Beta-Glycerophosphoric acid	707	758742	775162	-2.1	2
GC_68	37.36	1.86	80	Linolenic acid	847	774062	780147	-0.8	2
GC_69	37.56	1.78	117	Stearic acid	873	781603	787954	-0.8	1
GC_70	38.03	1.80	387	Glucose-6-phosphate	735	799327	817575	-2.2	2
GC_71	38.96	1.94	117	Arachidic acid	825	832360	855981	-2.8	1

GC_72	39.10	1.84	204	Cellobiose	777	926513	933726	-0.8	2
GC_73	40.36	1.94	437	Sucrose	806	891358	914209	-2.5	2
GC_74	41.16	2.12	204	Maltose	838	940962	946639	-0.6	2
GC_75	42.30	2.54	117	Lignoceric acid	879	986108	977654	0.9	2
GC_76	44.43	3.44	223	gamma-Tocopherol	787	1031876	n.a	n.a	2

Note:

RT, Retention time;

CV, Coefficient of variation. CV = (Calculated RI - Fiehn RI) / Fiehn RI

n.a, not available

Level 1 was achieved by commercial standard

Supplemental Table S3 Lettuce metabolites putatively identified by UPLC-IMS-QTOF-MS

No.	Component name	Neutral (*m/z*)	Observed (*m/z*)	Mass Error (ppm)	RT (min)	CCS (Å^2)	Adducts	Major fragments *m/z* (%)	Molecular Formula	Ref.	Level of identification
LC_77	1-(sn-glycero-3-phospho)-1D-myo-inositol	334.0665	333.0587	-1.6	0.68	162.87	M-H	241.0113(100); 92.9280(47); 154.0113(32); 259.0216(19)	C9H19O11P	Metlin	2
LC_78	Trisaccharide isomer 1[a]	504.1690	503.1615	-0.5	0.76	203.28	M-H	323.0981(22); 341.1082(6); 179.0554(9); 143.0345(2)	C18H32O16	Metlin	3
LC_79	Disaccharide isomer 1[b]	342.1158	341.1086	-1.1	0.77	169.05	M-H	179.055(22); 59.0131(22); 71.0139(10); 89.0245(7)	C12H22O11	Metlin	3
LC_80	UDP hexose isomer 1[c]	566.0550	565.0471	-1.1	0.86	206.66	M-H	323.0281(100); 384.9830(17); 272.9562(12)	C15H24N2O17P2	Metlin	3
LC_81	UDP hexose isomer 2[c]	566.0550	565.0474	-0.7	0.99	207.27	M-H	323.0278(100); 241.0119(11);	C15H24N2O17P2	Metlin	3
LC_82	Disaccharide isomer 2[b]	342.1162	341.1085	-1.3	1.06	169.52	M-H	59.0136(23); 179.0557(12); 71.0133(11)	C12H22O11	Metlin	3
LC_83	Disaccharide isomer 3[b]	342.1162	341.1083	-1.8	1.20	169.14	M-H	179.0549(8); 119.0350(2)	C12H22O11	Metlin	3
LC_84	Trisaccharide isomer 2[a]	504.1690	503.1616	-0.4	1.54	204.97	M-H	323.0977(34); 113.024(8); 179.0556(6); 161.0450(4); 89.0241(5)	C18H32O16	Metlin	3
LC_85	Cyanidin 3-O-galactoside	449.1084	449.1079	0.2	4.39	201.63	-e	287.0554(100)	C21H21O11	Standard	1
LC_86	Glutathione (oxidized form)	612.1520	611.1437	-1.5	4.45	216.74	M-H	306.0755(100); 272.0876(40); 254.0770(26); 210.0872(20)	C20H32N6O12S2	Metlin	2
LC_87	Guanosine	283.0917	282.0839	-1.9	4.50	158.10	M-H	133.0150(100); 150.04205(42);	C10H13N5O5	Metlin	2

								108.0196(16)			
LC_88	Xanthosine	284.0757	283.0680	-1.4	4.68	154.90	M-H	151.0254(100); 108.0197(30);	C10H12N4O6	Metlin	2
LC_89	L-Phenylalanine	165.0790	164.0714	-2.0	4.76	137.27	M-H	147.0450(86); 103.0555(12)	C9H11NO2	Metlin	2
LC_90	Dihydroxybenzoic acid	154.0266	153.0189	-1.2	4.77	168.87	M-H	109.0289(100)	C7H6O4	Metlin	3
LC_91	Cyanidin 3-(6"-malonylglucoside)	535.1088	535.1081	-0.2	4.86	215.35	-e	287.0559(100)	C24H23O14	ResPect	2
LC_92	Dihydrocaffeic acid hexose isomer 1	344.1107	343.1032	-0.7	4.93	165.78	M-H	163.0392(100); 181.0496(84); 119.0495(77); 135.0433(80)	C15H20O9	[1]	3
LC_93	Dihydroxybenzoic acid hexose isomer 1	316.0794	315.0718	-1.0	5.07	169.83	M-H	152.0112(31); 108.0216(10); 109.0280(20)	C13H16O9	[1]	3
LC_94	4-(2-hydroxyethyl) benzene-1,2-diol	154.0630	153.0552	-3.3	5.25	175.55	M-H	123.0443(100)	C8H10O3	In-house database	2
LC_95	Caffeoylquinic acid hexose isomer 1	516.1479	515.1411	-0.9	5.26	203.06	M-H	191.0556(100); 135.0453(5); 179.0347(4); 353.0876(3)	C22H28O14	[2]	3
LC_96	Dihydroxybenzoic acid hexose isomer 2	316.0794	315.0723	0.4	5.30	163.88	M-H	109.0291(100); 153.0189(39)	C13H16O9	[1]	3
LC_97	Caffeoyl-hexose isomer 1	342.0951	341.0883	1.5	5.54	177.16	M-H	135.0449(100); 179.0349(20)	C15H18O9	[3]	3
LC_98	Hydroxybenzoic acid hexose	300.0846	299.0773	0.1	5.54	162.30	M-H	137.0240(100)	C13H16O8	[1]	3
LC_99	Vanillic acid glucoside	330.0951	329.0877	-0.4	5.70	171.01	M-H	167.0345(100); 152.0105(19); 121.0294(10)	C14H18O9	[4]	2
LC_100	Caffeoylquinic acid hexose isomer 2	516.1479	515.1412	1.2	5.71	205.81	M-H	191.0558(100)	C22H28O14	[2]	3
LC_101	Esculetin hexoside isomer 1	340.0794	339.0721	-0.1	5.75	172.34	M-H	177.0185(100); 133.0289(15)	C15H16O9	ResPect	3
LC_102	Dihydrocaffeic acid hexose isomer 2	344.1107	343.1038	0.4	5.77	177.11	M-H	181.0500(100); 135.0447(90)	C15H20O9	[1]	3

LC_103	Geniposide	388.1369	387.1297	0.0	5.83	176.95	M-H	165.0554(33); 225.0769(100); 121.0291(34)	C17H24O10	[5]	2
LC_104	Quercetin hexoside glucuronide isomer 1	640.1276	639.1196	-1.1	5.94	235.10	M-H	463.0882(100); 300.0272(35)	C27H28O18	ResPect	3
LC_105	Isopropylmalic acid	176.0685	175.0608	-2.2	5.97	129.12	M-H	115.0399(100); 113.0608(13); 85.0655(8)	C7H12O5	[6]	2
LC_106	Dihydrocaffeic acid hexose isomer 3	344.1107	343.1029	-1.6	5.99	165.78	M-H	137.0607(100); 181.0502(78); 119.0495(29)	C15H20O9	[1]	3
LC_107	Quercetin hexoside glucuronide isomer 2	640.1276	639.1195	-1.2	6.00	249.44	M-H	463.0883(100); 301.0336(33); 300.0268(24); 271.0240(15)	C27H28O18	ResPect	3
LC_108	Quercetin 3, 4’-di-glucoside	626.1483	625.1404	-1.0	6.02	244.34	M-H	300.0262(13); 463.0879(100); 462.0801(35); 464.0909(22)	C27H30O17	Standard	1
LC_109	Caffeoyl-hexose isomer 2	341.0951	341.0876	-0.6	6.03	175.53	M-H	135.0449(100); 179.0347(20); 96.9599(13)	C15H18O9	[3]	3
LC_110	5-Caffeoylquinic acid (Caffeoylquinic acid isomer 1)[d]	354.0951	353.0877	-0.2	6.10	171.52	M-H	191.0558(100)	C16H18O9	Standard	1
LC_111	p-Coumaroyl glucoside	326.1002	325.0920	-1.6	6.12	167.90	M-H	119.0502(15); 163.0397(100)	C15H18O8	[7]	2
LC_112	Quercetin 3-O-(6"-O-malonyl)-glucoside 7-O-glucuronide	726.1280	725.1200	-1.0	6.19	243.85	M-H	505.0987(100); 300.0271(21); 301.0338(13)	C30H30O21	[8]	2
LC_113	Quercetin 3-O-(6"-O-malonyl)-glucoside 7-O-glucoside	712.1487	711.1407	-1.0	6.25	234.29	M-H	667.1510(100); 462.0804(59); 301.0345(38);	C30H32O20	Standard	1
LC_114	Esculetin hexoside isomer 2	340.0794	339.0723	0.6	6.31	166.90	M-H	177.0187(100)	C15H16O9	Metlin	3
LC_115	Caffeoyl-hexose isomer 3	342.0951	341.0879	0.3	6.47	169.55	M-H	135.0449(100); 179.0341(24);	C15H18O9	[3]	3

								96.9597(20)			
LC_116	4-Caffeoylquinic acid (Caffeoylquinic acid isomer 2)	354.0951	353.0877	-0.4	6.54	168.73	M-H	191.0554(65); 137.0241(6)	C16H18O9	[9]	1[e]
LC_117	Luteolin glucuronide-hexoside	624.1327	623.1244	-1.5	6.73	226.88	M-H	285.0398(100); 287.0563(27)	C27H28O17	[10]	2
LC_118	5-p-coumaroylquinic acid (p-coumaroylquinic acid isomer 1)	338.1002	337.0926	-0.9	6.73	187.16	M-H	191.0556(100); 93.0341(8)	C16H18O8	[9, 11]	1[e]
LC_119	Luteolin pentosyl-hexoside isomer 1	580.1428	625.1402	-1.3	6.76	225.12	M+FA-H	285.0398(100); 151.0034(28); 287.0563(27); 192.0596(20);	C26H28O15	ResPect	3
LC_120	Quercetin hexoside glucuronide isomer 3	640.1276	639.1198	-0.8	6.88	232.46	M-H	463.0878(20); 301.0343(100)	C27H28O18	ResPect	3
LC_121	Caffeoylmalic acid	296.0532	295.0455	-1.5	6.89	219.31	M-H	135.0453(100); 179.0347(92); 115.0036(81)	C13H12O8	[8]	2
LC_122	Luteolin diglucoside	610.1534	609.1456	-0.3	7.09	228.10	M-H	285.0399(100)	C27H30O16	ResPect	2
LC_123	15-deoxylactucin-8-sulfate isomer 1[f]	340.0617	339.0536	-2.5	7.11	169.11	M-H	96.96002(90)	C15H16O7S	[12]	2
LC_124	p-coumaroylquinic acid isomer 2	338.1002	337.0926	-1.0	7.13	167.56	M-H	191.0557(100)	C16H18O8	[13]	3
LC_125	Quercetin 3-neohesperidoside	610.1534	609.1457	-0.7	7.19	230.57	M-H	301.0339(100); 271.0241(16); 255.0291(8);	C27H30O16	ResPect	2
LC_126	Luteolin pentosyl-hexoside isomer 2	580.1428	579.1345	-1.8	7.42	224.12	M-H	285.0399(100); 461.0513(10)	C26H28O15	ResPect	3
LC_127	Luteolin 7-neohesperidoside	594.1585	593.1502	-1.6	7.43	227.35	M-H	285.0401(100)	C27H30O15	ResPect	2

LC_128	Quercetin 3-rutinoside (Rutin)	610.1514	609.1446	-2.5	7.43	230.57	M-H	301.0338(66); 300.0265(65)	C27H30O16	Standard	1
LC_129	(+)-5,5'-Dimethoxy-9-O-beta-D-glucopyranosyl lariciresinol	582.2312	581.2236	-0.7	7.48	227.54	M-H	329.1396(100); 341.1387(13)	C28H38O13	[12]	2
LC_130	Apigenin diglucoside	594.1573	593.1499	-2.1	7.54	234.31	M-H	269.0453(100)	C27H30O15	[14]	2
LC_131	Chicoric acid	474.0798	473.0724	-0.1	7.55	198.82	M-H	135.0448(100); 179.0345(64); 149.0088(34); 219.0293(15); 134.0366(9)	C22H18O12	Standard	1
LC_132	Quercetin 3-glucuronide	478.0747	477.0676	0.4	7.62	200.42	M-H	301.0348(100); 151.0031(15)	C21H18O13	Standard	1
LC_133	Quercetin 3-glucoside	464.0955	463.0878	-0.8	7.63	199.02	M-H	301.0348(100); 300.0269(35); 271.0239(21)	C21H20O12	Standard	1
LC_134	Luteolin 7-glucoside	448.1006	447.0933	0.2	7.65	209.25	M-H	285.0424(100)	C21H20O11	Standard	1
LC_135	Luteolin 7-glucuronide	462.0800	461.0727	0.2	7.67	194.05	M-H	285.0399(100)	C21H18O12	Standard	1
LC_136	Chicoric acid (isomer 2)	474.0798	473.0725	-0.1	7.69	202.18	M-H	135.0449(100); 179.0348(56); 149.0088(38); 219.0293(17); 161.0241(10); 191.0342(8)	C22H18O12	[8]	2
LC_137	Mono-hydroxylated dicaffeoylquinic acid	532.1208	531.1135	-0.9	7.80	205.55	M-H	191.0556(100)	C25H24O13	[15]	3
LC_138	Quercetin 3-(6"-malonylglucoside)	550.0959	549.0880	-0.6	7.82	216.03	M-H	505.0988(100); 300.0271(48)	C24H22O15	Standard	1
LC_139	Quercetin hexoside (Quercetin glucoside isomer 2)	464.0953	463.0880	-0.4	7.85	198.90	M-H	300.0271(100); 271.0243(41); 255.0292(22)	C21H20O12	ResPect	3
LC_140	8-deacetylmatricarin-8-sulfate[f]	342.0773	341.0697	-0.4	7.87	170.67	M-H	96.9600(100)	C15H18O7S	[16]	2

LC_141	Quercetin 3-glucoside -6”-acetate (isomer 1)	506.1060	505.0990	0.4	7.91	209.67	M-H	300.0271(100); 301.0336(48)	C23H22O13	ResPect	3
LC_142	Syringaresinol-glucoside	580.2156	579.2079	-0.4	7.94	234.54	M-H	417.1552(100)	C28H36O13	[17]	2
LC_143	15-deoxylactucin-8-sulfate isomer 2[f]	340.0617	339.0541	-0.3	7.97	169.11	M-H	96.9599(100)	C15H16O7S	[13]	2
LC_144	Quercetin 3-glucoside -6”-acetate (isomer 2)	506.1060	505.0991	0.6	8.05	212.01	M-H	300.0272(100)	C23H22O13	ResPect	3
LC_145	3,5-Dicaffeoyl quinic acid	516.1268	515.1194	-0.1	8.05	208.13	M-H	353.0883(13); 191.0558(100); 135.0446(39)	C25H24O12	[9]	1[e]
LC_146	Luteolin hexoside (isomer 2)	448.1006	447.0933	0	8.05	198.07	M-H	285.0398(100); 243.0298(66); 135.0452(55); 227.0347(33)	C21H20O11	ResPect	3
LC_147	Apigenin 7-O-glucoside	432.1057	431.0987	0.7	8.16	205.79	M-H	268.0374(100); 269.0439(43)	C21H20O10	Standard	1
LC_148	Apigenin 7-O-glucuronide	446.0849	445.0779	-0.6	8.22	202.77	M-H	269.0449(100)	C21H18O11	ResPect	2
LC_149	Quercetin diacetyl-hexoside	548.1166	547.1097	0.7	8.45	226.40	M -H	301.0333(100); 505.0987(14)	C25H24O14	[18]	3
LC_150	Lactucin[f]	276.0998	275.0923	-0.6	8.71	201.69	M -H	213.0917(100); 185.0967(13)	C15H16O5	[13]	2
LC_151	Lactucopicrin isomer 1[f]	410.1366	409.1295	0.6	8.94	203.60	M-H	213.0920(100); 257.0813(30);	C23H22O7	[13]	3
LC_152	Lactucopicrin isomer 2[f]	410.1366	409.1291	-0.4	9.30	199.85	M-H	213.0917(100); 151.0405(11)	C23H22O7	[13]	3
LC_153	Lactucopicrin-15-oxalate[f]	482.1211	481.1137	-0.7	9.43	199.52	M-H	213.0916(100); 185.0967(26); 257.0813(16)	C25H22O10	[13]	2
LC_154	Tri-4-hydroxyphenylacetyl glucoside isomer 1	582.1737	581.1658	-1.1	9.53	221.01	M-H	295.0814(100)	C30H30O12	[16]	2

LC_155	Tri-4-hydroxyphenylacetyl glucoside isomer 2	582.1737	581.1659	-1.0	9.62	218.65	M-H	295.0814(100); 143.0340(13)	C30H30O12	[16]	2
LC_156	Tri-4-hydroxyphenylacetyl glucoside isomer 3	582.1737	581.1658	-1.2	9.82	216.38	M-H	175.0397(100); 217.0500(52);	C30H30O12	[16]	2
LC_157	12-HpOTrE	310.2137	309.2064	-2.4	10.08	178.16	M-H	211.1335(100); 229.1440(60); 183.1387(27); 171.1022(19); 291.1859(14)	C18H30O4	[19]	2
LC_158	9S,12S,13S-trihydroxy-10E, 15Z-octadecadienoic acid	328.2250	327.2174	-0.3	10.08	182.51	M-H	229.1440(17); 283.0624(11); 233.1144(9); 171.1022(7); 212.1367(5)	C18H32O5	In-house database	3
LC_159	9,12,13-TriHOME	330.2407	329.2334	0.2	10.52	183.66	M-H	211.1338(100); 229.1439(54); 183.1390(26); 212.1370(11); 171.1024(9); 230.1472(6); 193.1220(6)	C18H34O5	In-house database	3
LC_160	Dodecanedioic acid	230.1505	229.1433	-5.9	10.52	152.32	M-H	211.1333(100)	C12H22O4	HMDB	3
LC_161	7E,9Z,11-Dodecatrienyl acetate	222.1602	221.1539	-3.6	12.24	182.04	M-H	220.1465(66); 177.0918(20); 148.0527(19); 192.1152(12)	C14H22O2	In-house database	3
LC_162	MGDG(18:5(3Z,6Z,9Z,12Z,15Z)/18:5(3Z,6Z,9Z,12Z,15Z))	766.4656	811.4666	3.7	13.29	279.51	M+FA-H	742.3920(100)	C45H66O10	Lipidmaps	3
LC_163	PE(16:0/0:0)	453.2855	452.2781	-0.3	13.72	212.79	M-H	255.2326(100)	C21H44NO7P	Lipidmaps	3
LC_164	27-nor-campestan-3beta,4beta,5alpha,6alpha,7beta,8beta,14alpha,15alpha,24-nonol	516.3298	561.3267	-2.7	13.93	236.61	M-H	480.3077(100)	C27H48O9	Lipidmaps	3
LC_165	PG(19:1 (9Z)/0:0)	524.3114	505.2958	4.2	14.00	221.99	M-H_2O-	152.9949(100)	C25H49O9P	Lipidmaps	3

							H				
LC_166	PA(16:0/18:2 (9Z,12Z))	672.4730	671.4623	-5.1	14.86	263.26	M-H	279.2325(100); 277.2170(89); 253.2160(5)	C37H69O8P	HMDB	3
LC_167	MGDG(20:5(5Z,8Z,11Z,14Z,17Z)/18:3(9Z,12Z,15Z))	798.5282	843.5247	-2.2	15.13	299.43	M+FA-H	742.4717(100); 241.0097(6)	C47H74O10	Lipidmaps	3
LC_168	PS(14:1 (9Z)/14:1 (9Z))	675.4111	720.4108	2.1	15.49	275.24	M+FA-H	537.2716(100)	C34H62NO10P	Lipidmaps	3
LC_169	PI (18:4 (6Z, 9Z, 12Z, 15Z) /0:0)	592.2649	591.2586	1.7	16.02	255.58	M-H	515.2427(100); 500.2180(7)	C27H45O12P	Lipidmaps	3
LC_170	MGDG (18:3 (9Z, 12Z, 15Z) / 18:3 (9Z, 12Z, 15Z))	774.5282	819.5254	-4.7	17.61	297.87	M+FA-H	277.2171(100); 513.3069(5)	C45H74O10	Lipidmaps	3
LC_171	MGDG (18:5(3Z, 6Z, 9Z, 12Z, 15Z) /18:4(6Z,9Z,12Z,15Z))	768.4812	813.4794	-0.1	17.84	288.67	M+FA-H	813.4784(100); 577.2680(3)	C45H68O10	Lipidmaps	3

Note: [a] consist of three molecules of aldohexoses; [b] consist of three molecules of aldohexoses; [c] the major forms are glucoside and galactoside. [d] common name is Chlorogenic acid; [e] identified by a surrogate standard from green coffee beans[20]; [f] validated by chicory and endive.

Supplemental Table S4 The volcano plot analysis of metabolites in leaf and head lettuce

No.	Name	Fold Change (FC)	log2(FC)	raw.pval	-log10(p)
LC_149	Quercetin diacetyl-hexoside	14.0810	3.8157	9.92E-12	11.0040
LC_103	Geniposide	9.1727	3.1973	5.75E-07	6.2401
LC_91	Cyanidin 3-(6"-malonylglucoside)	7.3238	2.8726	7.83E-05	4.1062
LC_144	Quercetin 3-glucoside -6"-acetate (isomer 2)	5.0893	2.3475	1.42E-13	12.8480
LC_113	Quercetin 3-O-(6"-O-malonyl)-glucoside 7-O-glucoside	4.0630	2.0226	1.31E-11	10.8810
LC_121	Caffeoylmalic acid	3.7293	1.8989	1.79E-04	3.7464
LC_85	Cyanidin 3-O-galactoside	3.6849	1.8816	1.26E-04	3.9012
LC_128	Quercetin 3-rutinoside (Rutin)	3.4468	1.7853	8.61E-07	6.0651
LC_111	p-Coumaroyl glucoside	3.2963	1.7208	1.22E-04	3.9136
LC_127	Luteolin 7-neohesperidoside	3.2619	1.7057	1.06E-03	2.9760
LC_95	Caffeoylquinic acid hexose isomer 1	3.2288	1.6910	5.98E-18	17.2230
LC_117	Luteolin glucuronide-hexoside	3.1286	1.6455	2.36E-05	4.6274
LC_141	Quercetin 3-glucoside -6"-acetate (isomer 1)	3.0946	1.6298	1.07E-13	12.9700
LC_122	Luteolin diglucoside	3.0704	1.6184	1.75E-10	9.7571
LC_125	Quercetin 3-neohesperidoside	2.9348	1.5533	1.11E-07	6.9536
LC_126	Luteolin pentosyl-hexoside isomer 2	2.8428	1.5073	5.29E-06	5.2761
LC_120	Quercetin hexoside glucuronide isomer 3	2.6560	1.4093	6.55E-09	8.1837
LC_112	Quercetin 3-O-(6"-O-malonyl)-glucoside 7-O-glucuronide	2.5374	1.3434	1.19E-09	8.9259
LC_135	Luteolin 7-glucuronide	2.5051	1.3249	1.14E-07	6.9434
LC_102	Dihydrocaffeic acid hexose isomer 2	2.4825	1.3118	4.26E-10	9.3711
LC_100	Caffeoylquinic acid hexose isomer 2	2.4770	1.3086	2.72E-15	14.5660
LC_145	3,5-Dicaffeoylquinic acid	2.4770	1.3086	3.83E-04	3.4168
GC_65	Caffeic acid	2.4758	1.3079	9.90E-06	5.0044
LC_90	Dihydroxybenzoic acid	2.3941	1.2595	8.11E-10	9.0909
LC_136	Chicoric acid (isomer 2)	2.3817	1.2520	2.38E-03	2.6240
LC_106	Dihydrocaffeic acid hexose isomer 3	2.3556	1.2361	2.49E-09	8.6034
LC_131	Chicoric acid	2.3477	1.2312	2.95E-04	3.5303
LC_97	Caffeoyl-hexose isomer 1	2.3016	1.2026	1.43E-05	4.8433
LC_108	Quercetin3, 4’-di-glucoside	2.2090	1.1434	2.54E-06	5.5948
LC_116	4-Caffeoylquinic acid (Caffeoylquinic acid isomer 2)	2.1826	1.1260	1.97E-03	2.7062
LC_132	Quercetin 3-glucuronide	2.0107	1.0077	1.43E-06	5.8445

Supplemental Table S5 The VIP scores of metabolites in leaf and head lettuce

No.	Name	Comp. 1	Comp. 2	Comp. 3	Comp. 4	Comp. 5
LC_95	Caffeoylquinic acid hexose isomer 1	2.5000	1.9135	1.7658	1.7078	1.6795
LC_100	Caffeoylquinic acid hexose isomer 2	2.3254	1.8359	1.6942	1.6385	1.6100
LC_141	Quercetin 3-glucoside -6"-acetate (isomer 1)	2.2078	1.7044	1.5634	1.5195	1.4904
LC_144	Quercetin 3-glucoside -6"-acetate (isomer 2)	2.1984	1.6906	1.5472	1.5006	1.4717
LC_94	4-(2-hydroxyethyl) benzene-1,2-diol	2.0963	1.7342	1.5853	1.5327	1.5033
LC_149	Quercetin diacetyl-hexoside	2.0467	1.5710	1.4427	1.3964	1.3795
LC_113	Quercetin 3-O-(6"-O-malonyl)-glucoside 7-O-glucoside	2.0360	1.6667	1.5211	1.4865	1.4580
LC_122	Luteolin diglucoside	1.9333	1.5509	1.4428	1.4112	1.3844
LC_118	5-p-coumaroylquinic acid (p-coumaroylquinic acid isomer 1)	1.9123	1.5679	1.4311	1.3879	1.3873
LC_102	Dihydrocaffeic acid hexose isomer 2	1.8961	1.4439	1.4202	1.3848	1.3583
LC_90	Dihydroxybenzoic acid	1.8684	1.4583	1.3655	1.3484	1.3444
LC_112	Quercetin 3-O-(6"-O-malonyl)-glucoside 7-O-glucuronide	1.8518	1.4890	1.3602	1.3480	1.3255
LC_106	Dihydrocaffeic acid hexose isomer 3	1.8188	1.4086	1.3916	1.3663	1.3523
LC_120	Quercetin hexoside glucuronide isomer 3	1.7746	1.3853	1.2764	1.2912	1.2787
LC_101	Esculetin hexoside isomer 1	1.7345	1.3433	1.2705	1.2704	1.2486
GC_75	Lignoceric acid	1.7105	1.5742	1.4874	1.4671	1.4391
GC_26	Glycine	1.6740	1.2759	1.2956	1.2688	1.2448
LC_125	Quercetin 3-neohesperidoside	1.6355	1.3693	1.3003	1.2851	1.2670
LC_135	Luteolin 7-glucuronide	1.6343	1.4224	1.3749	1.3290	1.3134
LC_124	p-coumaroylquinic acid isomer 2	1.6267	1.3569	1.2385	1.2322	1.2193
GC_63	Neophytadiene	1.5965	2.0456	1.8846	1.8232	1.8123
LC_103	Geniposide	1.5474	1.7780	1.6689	1.6135	1.5881
LC_115	Caffeoyl-hexose isomer 3	1.5378	1.1918	1.2669	1.2246	1.2067
LC_128	Quercetin 3-rutinoside (Rutin)	1.5248	1.3664	1.2489	1.2553	1.2316
GC_22	L-isoleucine	1.5051	1.1699	1.1419	1.1292	1.1086
GC_56	Lyxose	1.5003	1.6611	1.5183	1.4717	1.4629
LC_132	Quercetin 3-glucuronide	1.4957	1.3286	1.2402	1.2409	1.2398
LC_108	Quercetin3, 4’-di-glucoside	1.4619	1.3177	1.2071	1.1957	1.1897
GC_35	Threonine	1.4322	1.0967	1.1262	1.1048	1.0952
LC_126	Luteolin pentosyl-hexoside isomer 2	1.4174	1.5133	1.4332	1.3980	1.3730
GC_65	Caffeic acid	1.3782	1.0578	1.0650	1.0869	1.0738

LC_133	Quercetin 3-glucoside	1.3593	1.4083	1.4105	1.3661	1.3474
GC_14	Propanedioic acid	1.3590	1.0464	1.0215	0.9906	0.9879
LC_97	Caffeoyl-hexose isomer 1	1.3543	1.6831	1.6280	1.5781	1.5548
LC_117	Luteolin glucuronide-hexoside	1.3215	1.0706	0.9913	0.9592	1.0274
GC_15	L-valine	1.3144	1.0213	1.0567	1.0557	1.0427
LC_91	Cyanidin 3-(6"-malonylglucoside)	1.2384	0.9751	0.9112	0.9428	0.9499
GC_24	Maleic acid	1.2382	1.3407	1.3831	1.3680	1.3696
LC_98	Hydroxybenzoic acid hexose	1.2264	1.2975	1.2268	1.1863	1.1636
LC_111	p-Coumaroyl glucoside	1.2061	1.1984	1.2650	1.2258	1.2022
LC_85	Cyanidin 3-O-galactoside	1.2040	0.9407	0.8909	0.9365	0.9355

Supplemental Table S6 The Mean Decrease Accuracy of metabolites in leaf and head lettuce

No.	Name	Mean Decrease Accuracy
LC_100	Caffeoylquinic acid hexose isomer 2	1.95E-02
LC_117	Luteolin glucuronide-hexoside	1.70E-02
LC_95	Caffeoylquinic acid hexose isomer 1	1.53E-02
GC_63	Neophytadiene	1.42E-02
GC_56	Lyxose	1.41E-02
LC_144	Quercetin 3-glucoside -6"-acetate (isomer 2)	1.34E-02
LC_90	Dihydroxybenzoic acid	1.32E-02
LC_103	Geniposide	1.10E-02
LC_86	Glutathione (oxidized form)	1.09E-02
LC_102	Dihydrocaffeic acid hexose isomer 2	1.03E-02
LC_113	Quercetin 3-O-(6"-O-malonyl)-glucoside 7-O-glucoside	1.01E-02
GC_42	Asparagine	9.62E-03
LC_141	Quercetin 3-glucoside -6"-acetate (isomer 1)	8.37E-03
LC_122	Luteolin diglucoside	7.95E-03
LC_149	Quercetin diacetyl-hexoside	7.22E-03
GC_67	Beta-Glycerophosphoric acid	7.20E-03
LC_112	Quercetin 3-O-(6"-O-malonyl)-glucoside 7-O-glucuronide	7.12E-03
LC_120	Quercetin hexoside glucuronide isomer 3	6.91E-03
LC_143	15-deoxylactucin-8-sulfate isomer 2	6.90E-03
LC_119	Luteolin pentosyl-hexoside isomer 1	6.55E-03
GC_35	Threonine	6.47E-03
LC_167	MGDG(20:5(5Z,8Z,11Z,14Z,17Z)/18:3(9Z,12Z,15Z))	6.42E-03
GC_26	Glycine	6.32E-03
GC_22	L-isoleucine	6.31E-03
GC_62	Shikimic acid	5.82E-03
GC_41	Meso-Erythritol	5.82E-03
GC_38	Beta-Alanine	5.78E-03
GC_57	Ribitol	5.77E-03
LC_99	Vanillic acid glucoside	5.49E-03
LC_97	Caffeoyl-hexose isomer 1	5.38E-03
LC_147	Apigenin 7-O-glucoside	5.19E-03
LC_118	5-p-coumaroylquinic acid (p-coumaroylquinic acid isomer 1)	5.17E-03
LC_106	Dihydrocaffeic acid hexose isomer 3	5.11E-03

LC_140	8-deacetylmatricarin-8-sulfate	4.94E-03
GC_8	2-Hydroxybutyric acid	4.55E-03
LC_124	p-coumaroylquinic acid isomer 2	4.40E-03
LC_150	Lactucin	4.28E-03
LC_101	Esculetin hexoside isomer 1	4.27E-03
LC_135	Luteolin 7-glucuronide	4.10E-03
LC_156	Tri-4-hydroxyphenylacetyl glucoside isomer 3	4.10E-03

Supplemental Table S7 Characteristics of leaf and head lettuce networks

	Leaf	Head
Network description		
Average degree	3.053	2.496
Network diameter	5	7
Density	0.033	0.022
Modularity	0.602	0.614
Modules	Module I (27.66%)	Module I (21.24%)
	Module II (25.53%)	Module II (17.70%)
	Module III (10.64%)	Module III (12.39%)
	Module IV (10.64%)	Other modules [a] (48.67%)
	Other modules [a] (25.53%)	
Weakly connected components	11	16
Strongly connected components	94	113
Node description		
Total nodes	94	113
Node connectivity	2.519	2.067
Average clustering coefficient	0.263	0.206
Eigenvector centrality (sum change)	0.0124	0.0119
Edge description		
Total edges	287	282
Average path length	1.677	2.094

Note: [a] Other modules included all the module with less than 10% connections.

Supplemental Reference

1. Viacava, G. E. *et al.* Characterization of phenolic compounds in green and red oak-leaf lettuce cultivars by UHPLC-DAD-ESI-QToF/MS using MS^E scan mode. *J. Mass Spectrom.* **52**, 873-902 (2017).
2. Liao, S. *et al*. Rapid screening and identification of caffeic acid and its esters in *Erigeron breviscapus* by ultra-performance liquid chromatography/tandem mass spectrometry. *Rapid Commun. Mass Sp.* **24**, 2533-2541 (2010).
3. Amessis-Ouchemoukh, N. *et al.* Tentative characterisation of iridoids, phenylethanoid glycosides and flavonoid derivatives from *Globularia alypum* L. (globulariaceae) leaves by LC-ESI-QTOF-MS. *Phytochem. Analysis.* **25**, 389-398 (2014).
4. Fang, N. Yu, S. & Prior, R. L. LC/MS/MS characterization of phenolic constituents in dried plums. *J. Agr. Food Chem.* **50**, 3579-3585 (2002).
5. Wang, X. *et al*. Analysis of the constituents in the rat plasma after oral administration of Yin Chen Hao Tang by UPLC/Q-TOF-MS/MS. *J. Pharmaceut. Biomed.* **46**, 477-490 (2008).
6. Gómez-Romero, M. Segura-Carretero, A. & Fernández-Gutiérrez, A. Metabolite profiling and quantification of phenolic compounds in methanol extracts of tomato fruit. *Phytochemistry.* **71**, 1848-1864 (2010).
7. Seeram, N. P. Lee, R. Scheuller, H. S. & Heber, D. Identification of phenolic compounds in strawberries by liquid chromatography electrospray ionization mass spectroscopy. *Food Chem.* **97**, 1-11 (2006).
8. Llorach, R. Martínez-Sánchez, A. Tomás-Barberán, F. A. Gil, M. I. & Ferreres, F. Characterisation of polyphenols and antioxidant properties of five lettuce varieties and escarole. *Food Chem.* **108**, 1028-1038 (2008).
9. Clifford, M. N. Johnston, K. L. Knight, S. & Kuhnert, N. Hierarchical scheme for LC-MS^n identification of chlorogenic acids. *J. Agr. Food Chem.* **51**, 2900-2911 (2003).
10. Abu-Reidah, I. M. Arráez-Román, D. Segura-Carretero, A. & Fernández-Gutiérrez, A. Extensive characterisation of bioactive phenolic constituents from globe artichoke (*Cynara scolymus* L.) by HPLC-DAD-ESI-QTOF-MS. *Food Chem.* **141**, 2269-2277 (2013).
11. Clifford, M. N. Wu, W. Kirkpatrick, J. & Kuhnert, N. Profiling the chlorogenic acids and other caffeic acid derivatives of herbal Chrysanthemum by LC-MS^n. *J. Agr. Food Chem.* **55**, 929-936 (2007).
12. Abu-Reidah, I. M. Contreras, M. M. Arráez-Román, D. Segura-Carretero, A. & Fernández-Gutiérrez, A. Reversed-phase ultra-high-performance liquid chromatography coupled to electrospray ionization-quadrupole-time-of-flight mass spectrometry as a powerful tool for metabolic profiling of vegetables: *Lactuca sativa* as an example of its application. *J. Chromatogr. A.* **1313**, 212-227 (2013).
13. Sessa, R. A. Bennett, M. H. Lewis, M. J. Mansfield, J. W. & Beale, M. Metabolite profiling of sesquiterpene lactones from *Lactuca* species major latex components are novel oxalate and sulfate conjugates of lactucin and its derivatives. *J. Biol. Chem.* **275**, 26877-26884 (2000).
14. Pacheco-Palencia, L. A. Duncan, C. E. & Talcott, S. T. Phytochemical composition and thermal stability of two commercial açai species, *Euterpe oleracea* and *Euterpe precatoria*. *Food Chem.*

115, 1199-1205 (2009).

15. Santos, M. D. Lopes, N. P. & Iamamoto, Y. HPLC-ESI-MS/MS analysis of oxidized di-caffeoylquinic acids generated by metalloporphyrin-catalyzed reactions. *Quim. Nova.* **31**, 767-770 (2008).
16. García, C. J. García-Villalba R, Garrido Y, Gil M. I. & Tomás-Barberán, F. A. Untargeted metabolomics approach using UPLC-ESI-QTOF-MS to explore the metabolome of fresh-cut iceberg lettuce. *Metabolomics*. **12**, 138 (2016).
17. Sun, H. *et al*. Characterization of the multiple components of *Acanthopanax Senticosus* stem by ultra high performance liquid chromatography with quadrupole time-of-flight tandem mass spectrometry. *J. Sep. Sci.* **39**, 496-502 (2016).
18. Navarro-González, I. González-Barrio, R. García-Valverde, V. Bautista-Ortín, A. B. & Periago, M. J. Nutritional composition and antioxidant capacity in edible flowers: characterisation of phenolic compounds by HPLC-DAD-ESI/MS^n. *Int. J. Mol. Sci.* **16**, 805-822 (2014).
19. Sooman, L. & Oliw, E. H. Discovery of a novel linoleate dioxygenase of *Fusarium oxysporum* and linoleate diol synthase of *Colletotrichum graminicola*. *Lipids.* **50**, 1243-1252 (2015).
20. Clifford, M. N. & Madala N. D. Surrogate standards: a cost-effective strategy for identification of phytochemicals. *J. Agric. Food Chem.* **65**, 3589-3590 (2017).
