# Supplementary material for: A novel integrated non-targeted metabolomic analysis reveals significant metabolite variations between different lettuce (Lactuca sativa. L) varieties
Source: Hortic Res. 2018 Jun 25;5:33. doi: 10.1038/s41438-018-0050-1 (PMC6015802; doi:10.1038/s41438-018-0050-1)
Supplement: Supplementary file 2 — Supplemental Figures [file 41438_2018_50_MOESM2_ESM.pdf]

Supplemental Figure Captions

Supplemental Figure S1. Phenotypes of 30 lettuce varieties cultivated in field

Supplemental Figure S2. 3D GC×GC/TOF-MS and UPLC-IMS-QTOF/MS chromatography of lettuce extraction

Note: A, GC×GC/TOF-MS; B, UPLC-IMS-QTOF/MS

Supplemental Figure S3 K-means clustering analysis of the metabolites of 30 cultivars of lettuce

Note: Distance measured by Euclidean and ward clustering algorithm

Supplemental figure S4 Random Forest Classification of leaf and head lettuces

Note: Cumulative error rates by Random Forest classification. The overall error rate is shown as the red line; the blue (leaf) and green (head) lines represent the error rates for each class

Supplemental figure S5 Heatmaps of the relative content of different metabolites in butterhead, iceberg and romaine lettuce

For amino acids; GABA, 4-Aminobutanoic acid;

For carbohydrates; G-1-P, Glucose-1-phosphate; Glycerol-2-P, Glucose-1-phosphate; Glycerol-2-P, Beta-Glycerophosphoric acid; G-6-P, Glucose-6-phosphate; 1-G-P-MI, 1-(sn-Glycero-3-phospho)-1D-myo-inositol; Trisaccharide 1, Trisaccharide isomer 1; Trisaccharide 2, Trisaccharide isomer 2; Disaccharide 1, Disaccharide isomer 1; Disaccharide 2, Disaccharide isomer 2; Disaccharide 3, Disaccharide isomer 3;

For Nucleotide and derivatives; UDP hexose 1, UDP hexose isomer 1; UDP hexose 2, UDP hexose isomer 2;

For organic acids; 2-Hydroxybutyrate, 2-Hydroxybutyric acid; Carboxylic acid, 3-Hydroxypropionic acid; 3-Hydroxybutyrate, 3-Hydroxybutyric acid; SSA, Succinate semialdehyde; GHB, 4-Hydroxybutanoic acid; Minaline, Pyrrole-2-carboxylic acid; m-Salicylate, 3-Hydroxybenzoic acid; 2-Ketoglutarate, Alpha-ketoglutaric acid; Isopropylmalate, Isopropylmalic acid;

For other compounds; DMC-sulfate, 8-deacetylmatricarin-8-sulfate; GSSG, Glutathione (oxidized form); glycerone, Dihydroxyacetone.

Supplemental Figure S6 Network analysis of leaf and head lettuce metabolites based on modularity.

Note: A, leaf lettuce network; B, head lettuce network

Supplemental figure S7 PCA analysis based on phenotypic features and original sources of 30 lettuce accessions

Note: Different color circles display 95% confidence regions of respective color groups. A, PCA

scores of leaf color; B, PCA scores of leaf shape, C, PCA scores of leaf texture, D, PCA scores of original lettuce sources, E, PCA scores of two groups

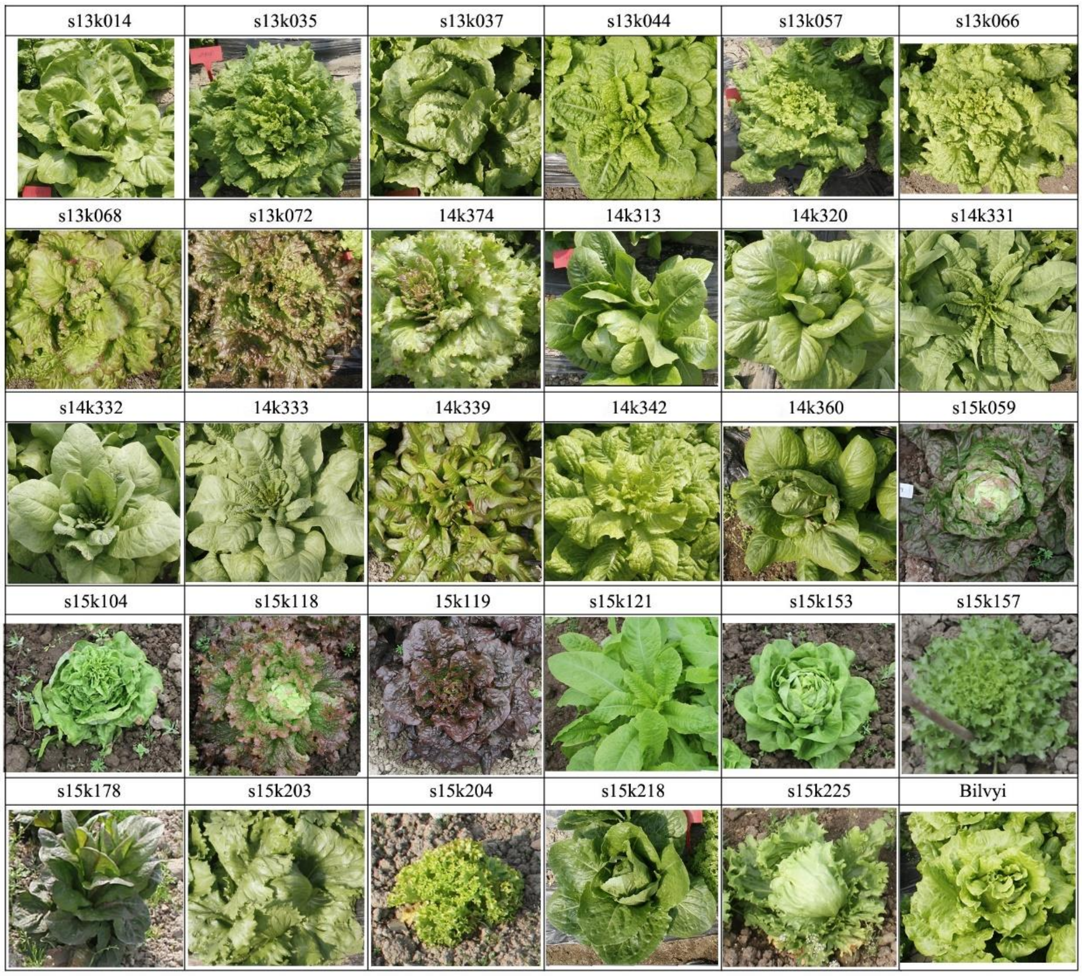

Supplemental Figure S1. Phenotypes of 30 lettuce varieties cultivated in field

A

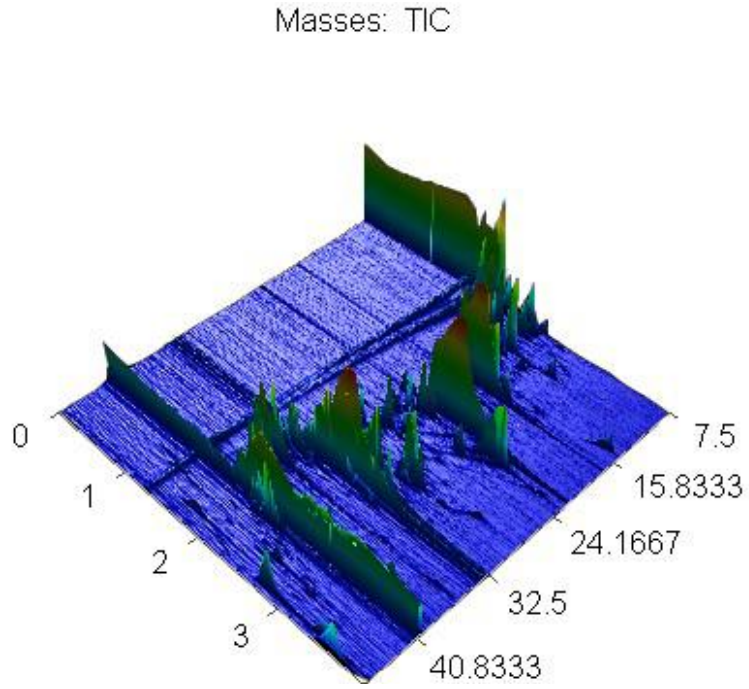

B

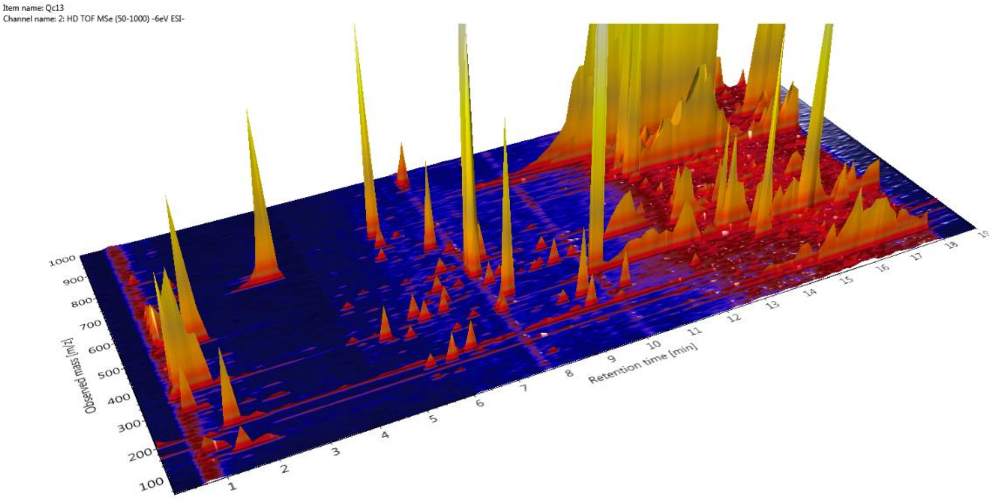

Supplemental Figure S2. 3D GC×GC/TOF-MS and UPLC-IMS-QTOF/MS chromatography of lettuce extraction

Note: A, GC×GC/TOF-MS; B, UPLC-IMS-QTOF/MS

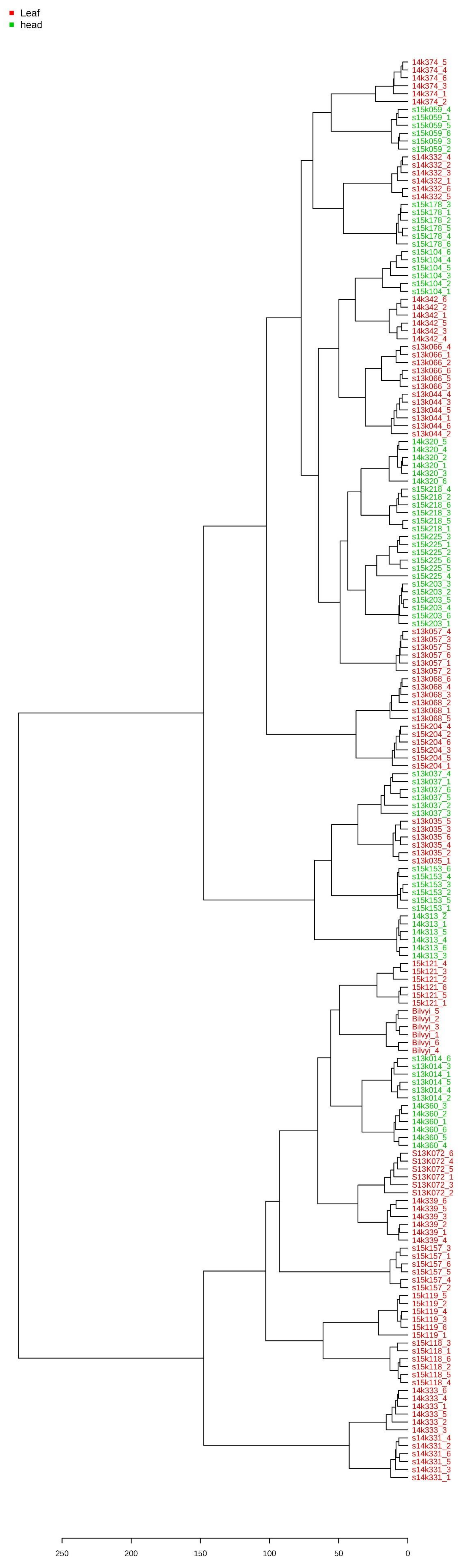

Supplemental Figure S3 K-means clustering analysis of 30 cultivars of lettuce

Note: Distance measured by Euclidean and ward clustering algorithm

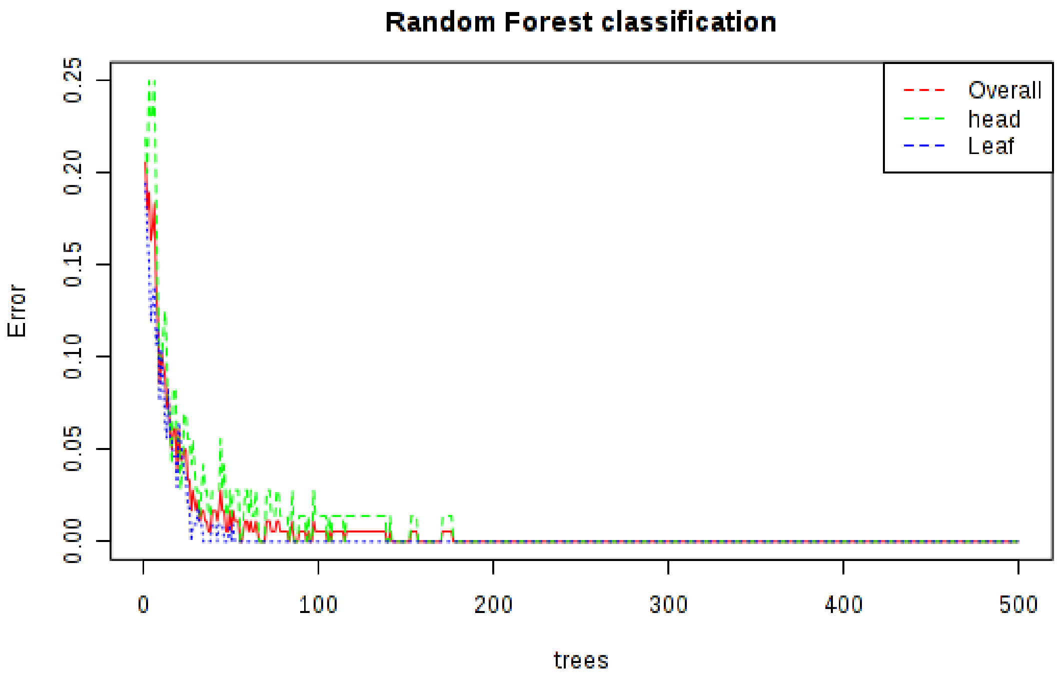

Types	leaf	head	classification error
leaf	108	0	0
head	0	72	0

Supplemental figure S4 Random Forest Classification of leaf and head lettuces

Note: Cumulative error rates by Random Forest classification. The overall error rate is shown as the red line; the blue (leaf) and green (head) lines represent the error rates for each class

Amino acids

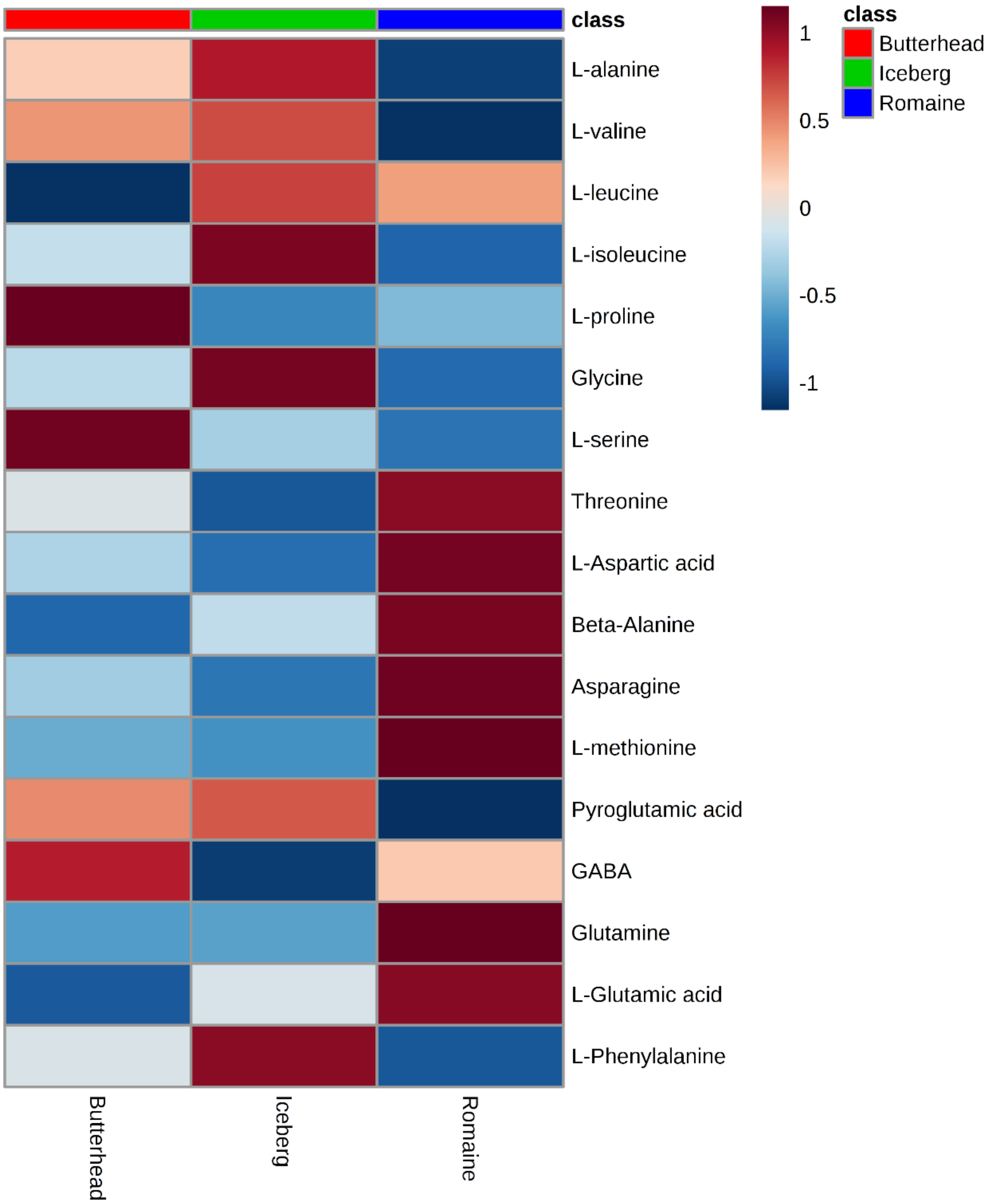

class
L-alanine
L-valine
L-leucine
L-isoleucine
L-proline
Glycine
L-serine
Threonine
L-Aspartic acid
Beta-Alanine
Asparagine
L-methionine
Pyroglutamic acid
GABA
Glutamine
L-Glutamic acid
L-Phenylalanine
Butterhead
Iceberg
Romaine
1
0.5
0
-0.5
-1
class
Butterhead
Iceberg
Romaine

Carbohydrates

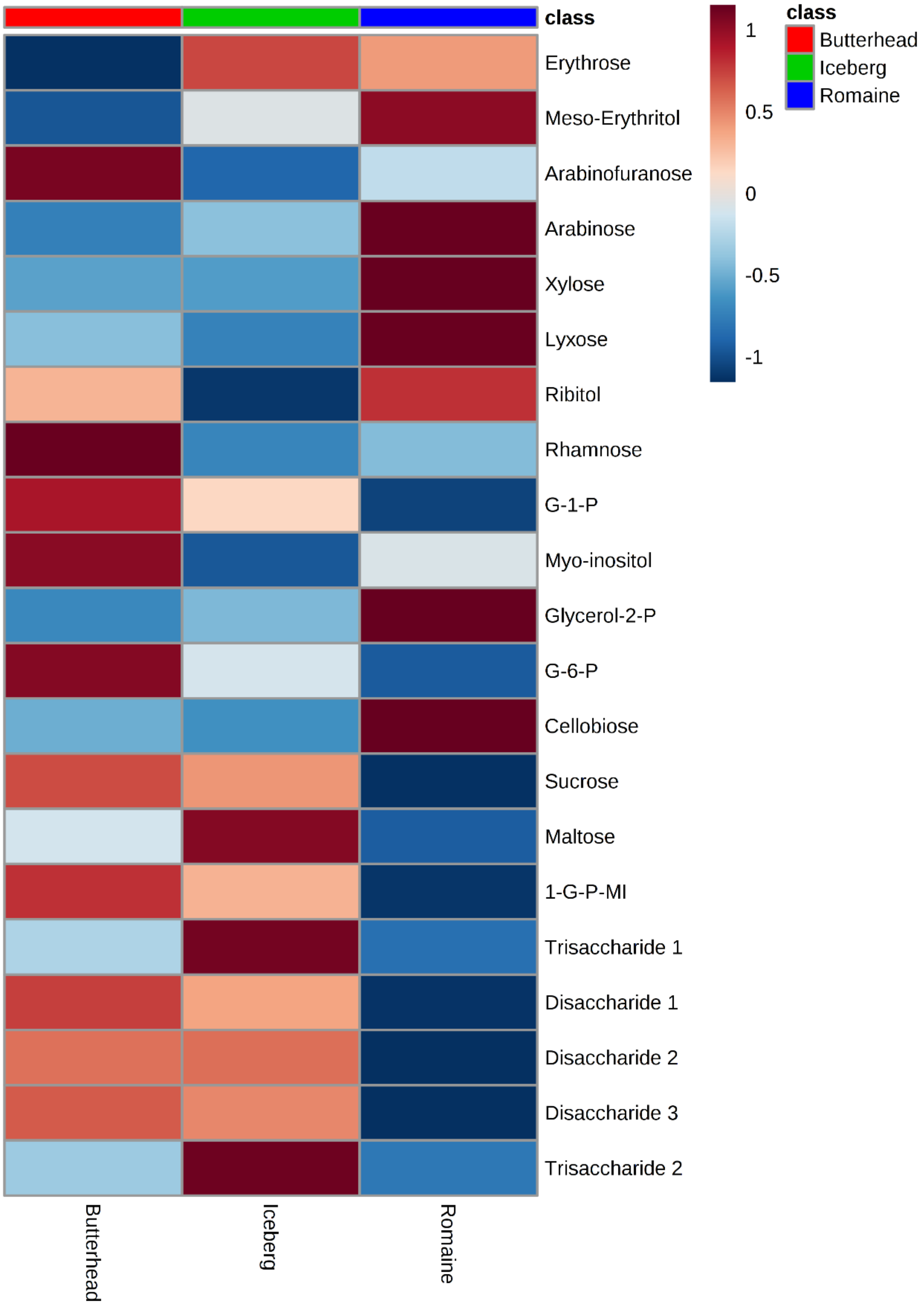

Nucleotide and derivatives

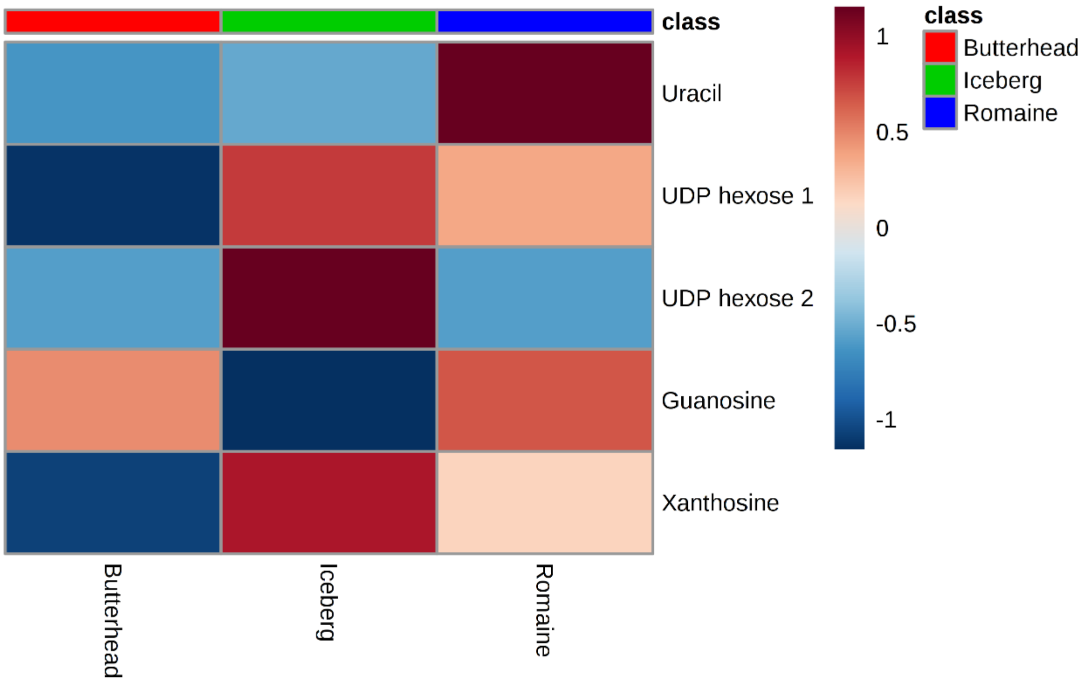

Organic acids

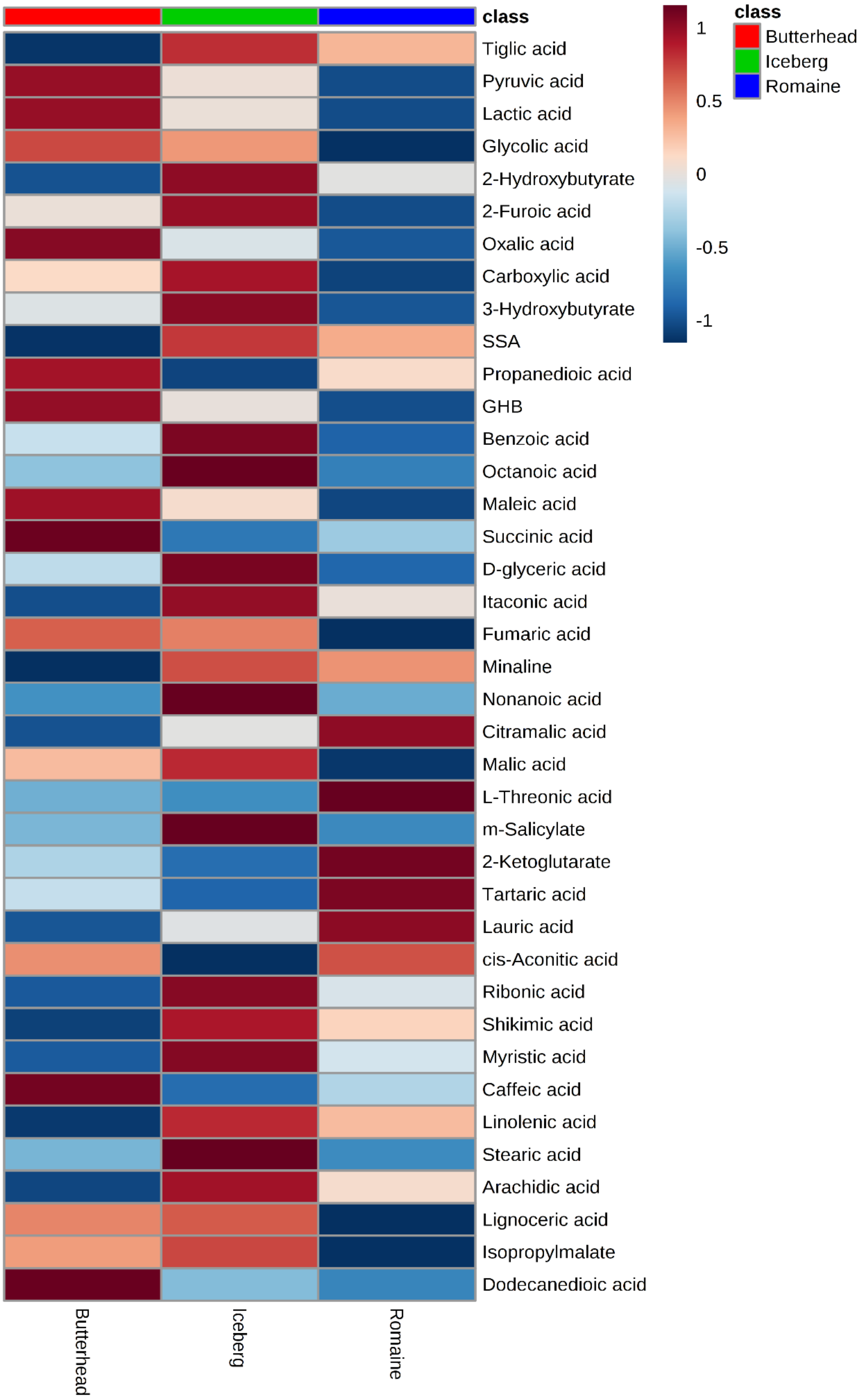

Others compounds

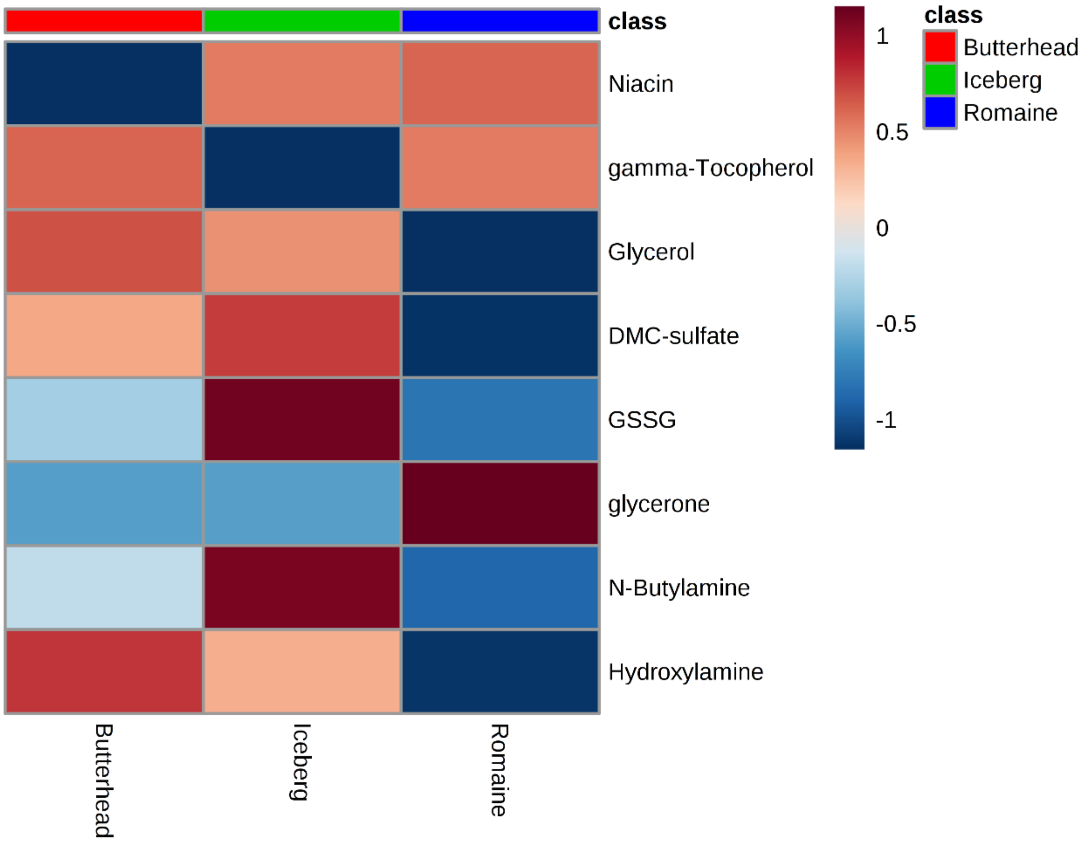

Supplemental figure S5 Heatmaps of the relative content of different metabolites in butterhead, iceberg and romaine lettuce

For carbohydrates; G-1-P, Glucose-1-phosphate; Glycerol-2-P, Glucose-1-phosphate; Glycerol-2-P, Beta-Glycerophosphoric acid; G-6-P, Glucose-6-phosphate; 1-G-P-MI, 1-(sn-Glycero-3-phospho)-1D-myo-inositol; Trisaccharide 1, Trisaccharide isomer 1; Trisaccharide 2, Trisaccharide isomer 2; Disaccharide 1, Disaccharide isomer 1; Disaccharide 2, Disaccharide isomer 2; Disaccharide 3, Disaccharide isomer 3;

For Nucleotide and derivatives; UDP hexose 1, UDP hexose isomer 1; UDP hexose 2, UDP hexose isomer 2;

For organic acids; 2-Hydroxybutyrate, 2-Hydroxybutyric acid; Carboxylic acid, 3-Hydroxypropionic acid; 3-Hydroxybutyrate, 3-Hydroxybutyric acid; SSA, Succinate semialdehyde; GHB, 4-Hydroxybutanoic acid; Minaline, Pyrrole-2-carboxylic acid; m-Salicylate, 3-Hydroxybenzoic acid; 2-Ketoglutarate, Alpha-ketoglutaric acid; Isopropylmalate, Isopropylmalic acid;

For other compounds; DMC-sulfate, 8-deacetylmatricarin-8-sulfate; GSSG, Glutathione (oxidized form); glycerone, Dihydroxyacetone.

(a) Leaf

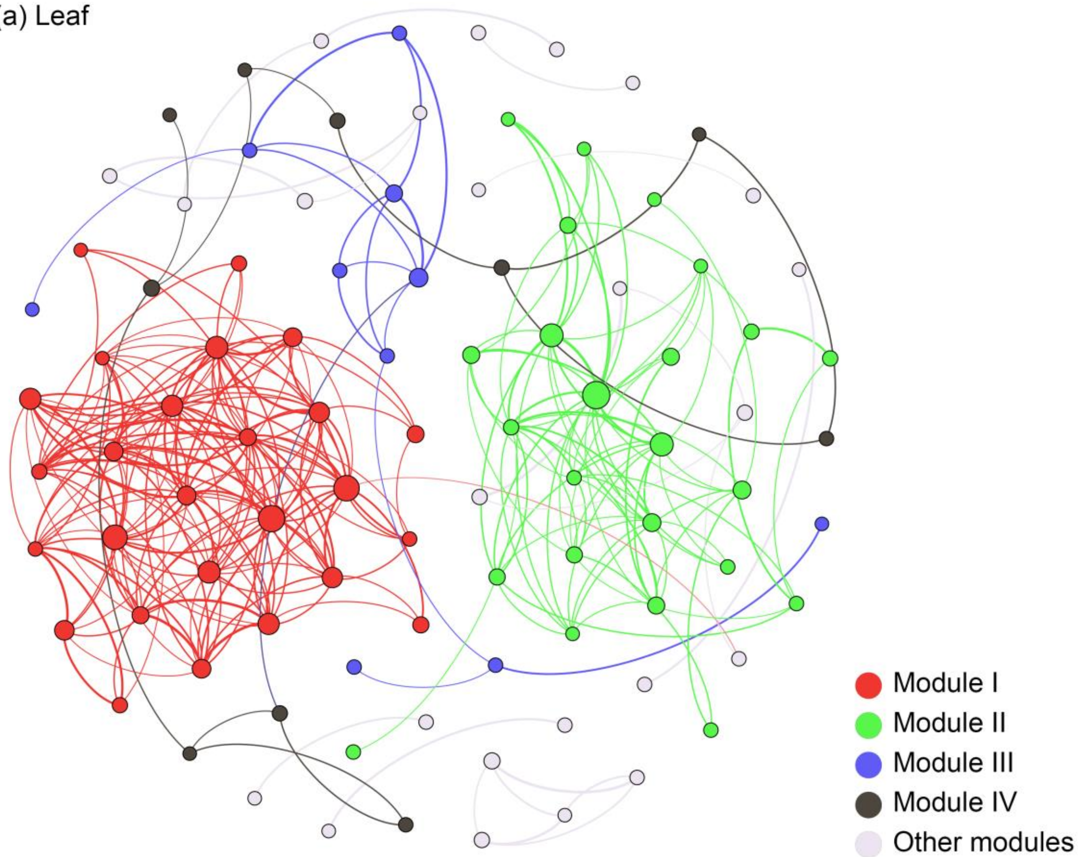

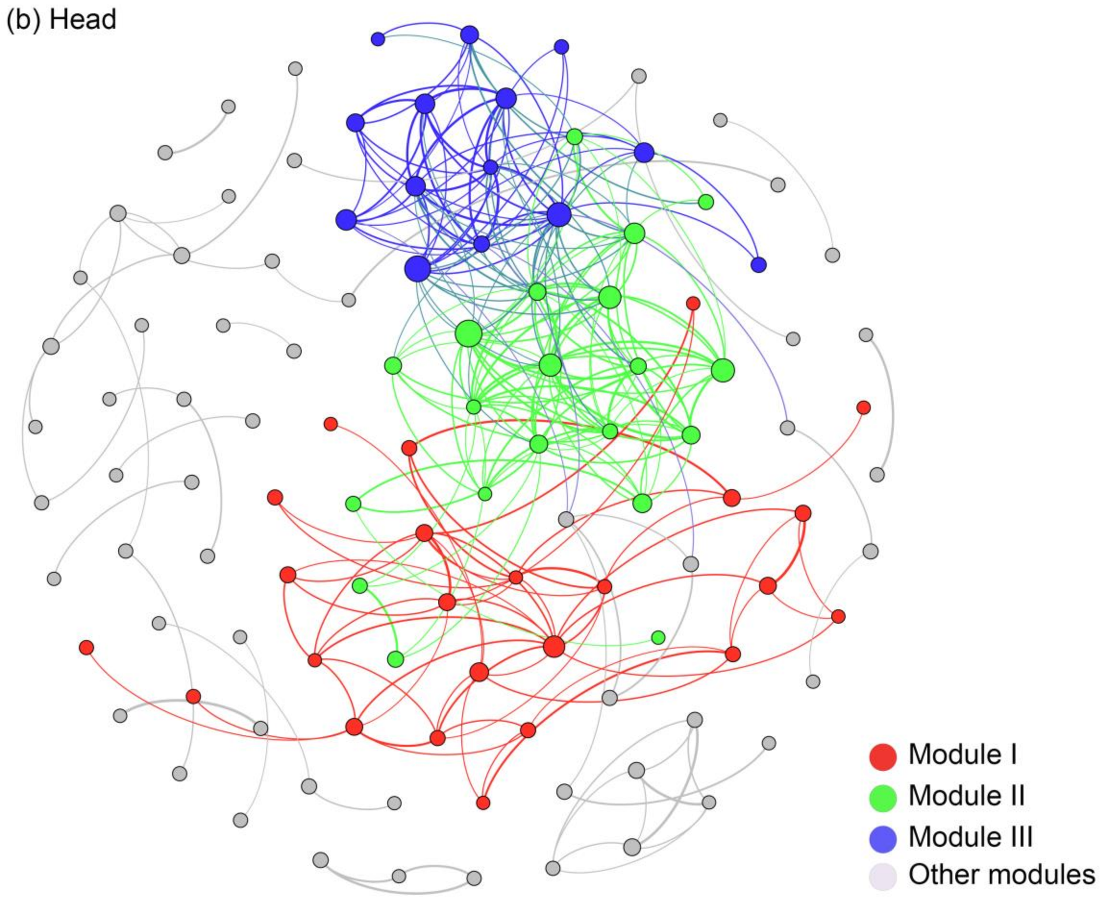

Supplemental Figure S6 Network analysis of leaf and head lettuce metabolites based on modularity.

Note: A, leaf lettuce network; B, head lettuce network

A

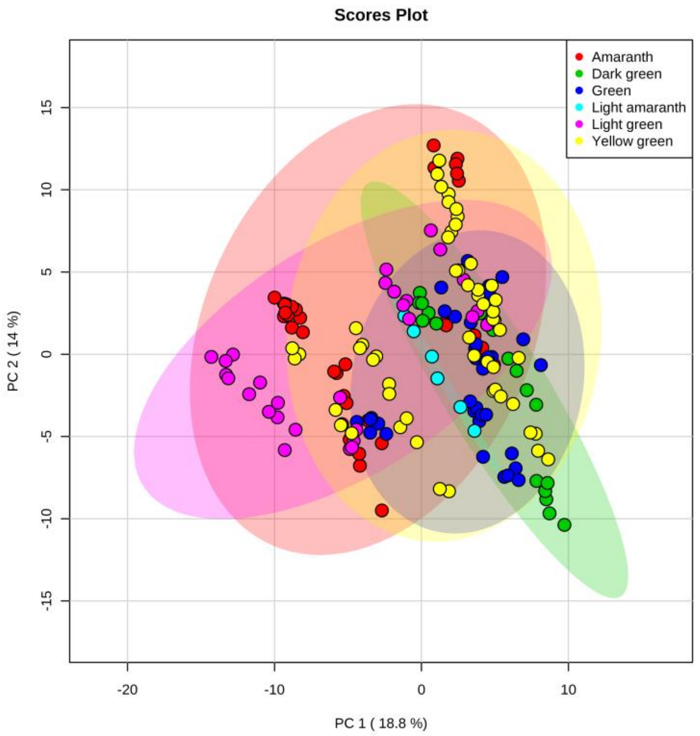

Scores Plot
Amaranth
Dark green
Green
Light amaranth
Light green
Yellow green
PC 2 (14 %)
PC 1 (18.8 %)

B

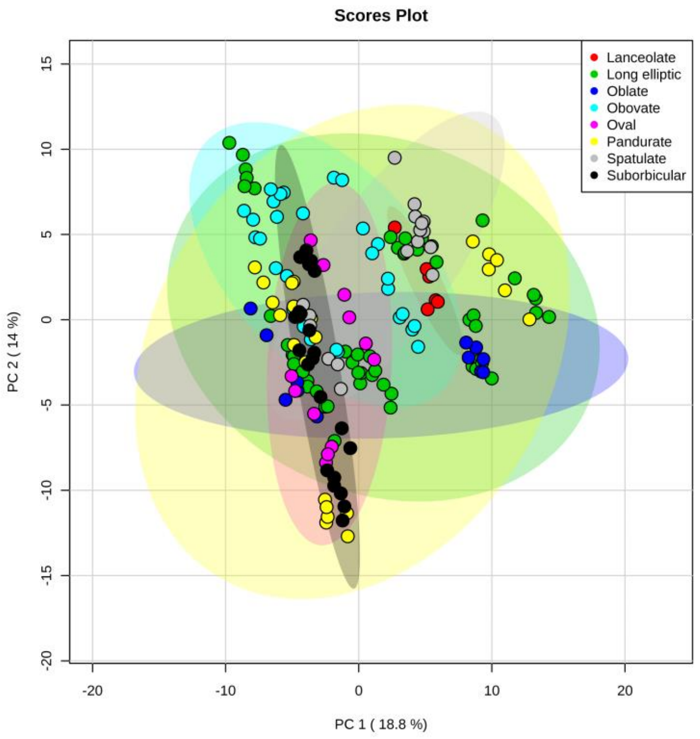

Scores Plot
Lanceolate
Long elliptic
Oblate
Obovate
Oval
Pandurate
Spatulate
Suborbicular
PC 2 (14 %)
PC 1 (18.8 %)

C

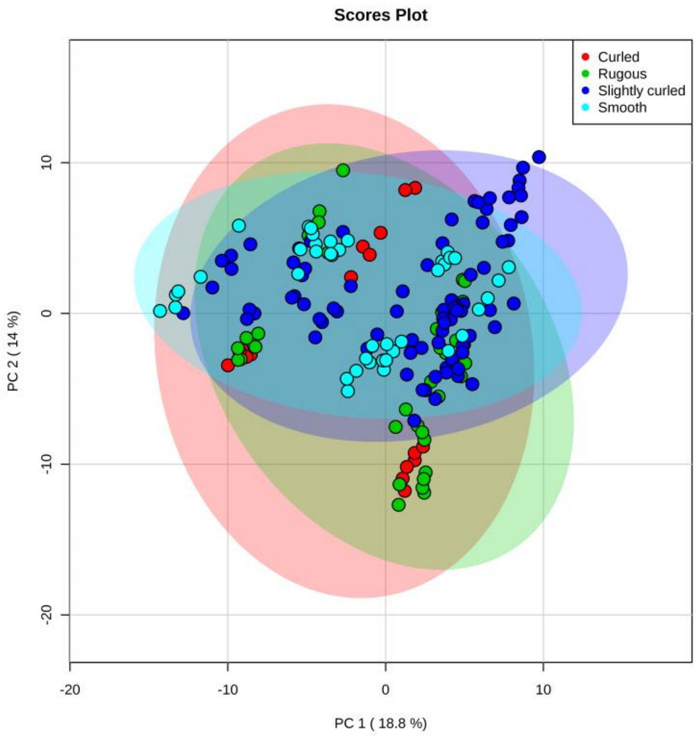
Scores Plot
Curled
Rugous
Slightly curled
Smooth
PC 2 (14 %)
PC 1 (18.8 %)
10
0
-10
-20
-20
-10
0
10

D

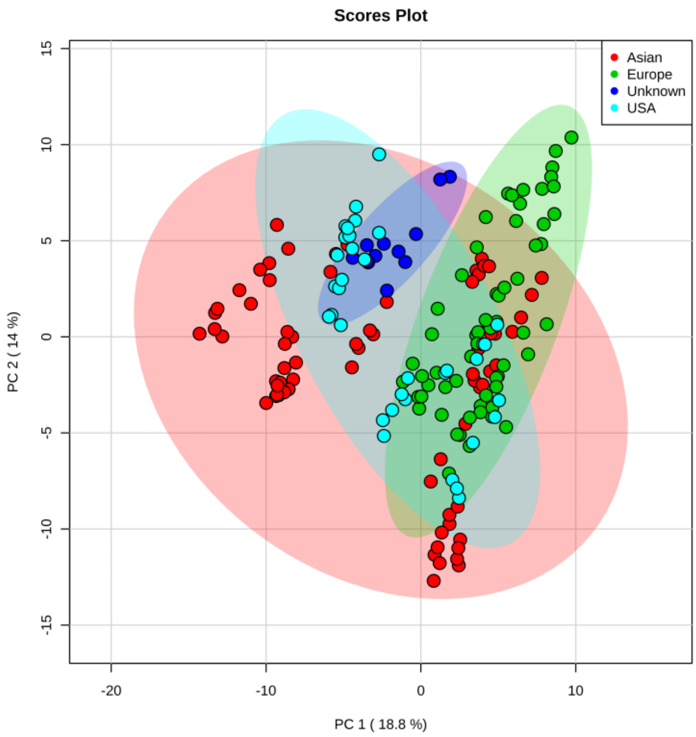
Scores Plot
Asian
Europe
Unknown
USA
PC 2 (14 %)
PC 1 (18.8 %)
15
10
5
0
-5
-10
-15
-20
-10
0
10

E

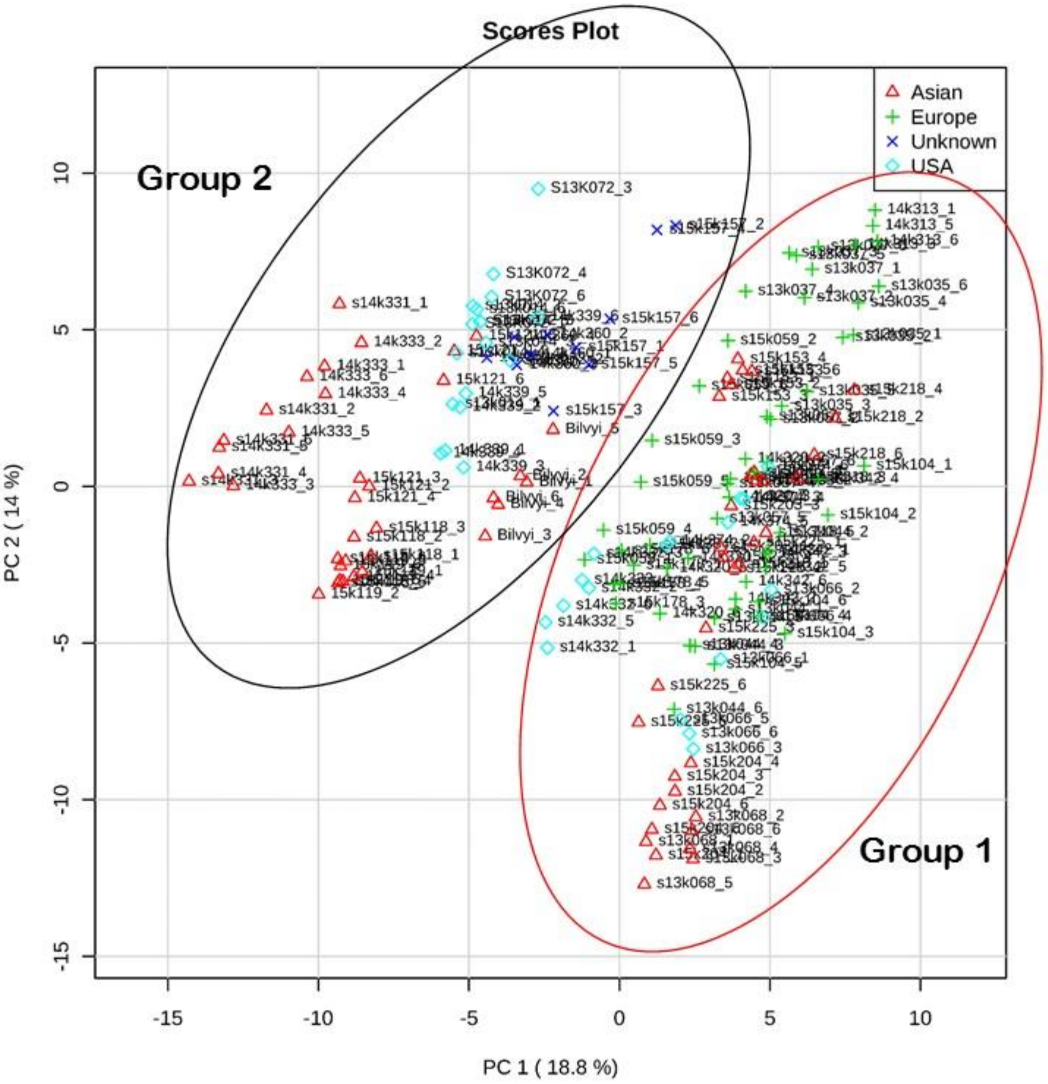

Supplemental figure S7 PCA analysis based on phenotypic features and original sources of 30 lettuce accessions

Note: Different color circles display 95% confidence regions of respective color groups. A, PCA scores of leaf color; B, PCA scores of leaf shape, C, PCA scores of leaf texture, D, PCA scores of original lettuce sources, E, PCA scores of two groups
